# Altered genome-wide hippocampal gene expression profiles following early life lead exposure and their potential for reversal by environmental enrichment

**DOI:** 10.1038/s41598-022-15861-9

**Published:** 2022-07-25

**Authors:** G. Singh, V. Singh, T. Kim, A. Ertel, W. Fu, J. S. Schneider

**Affiliations:** 1 Department of Pathology, Anatomy, and Cell Biology, Thomas Jefferson University, 1020 Locust Street, JAH 511, Philadelphia, PA USA; 2 Kimmel Cancer Center Department of Cancer Biology, Cancer Genomics and Bioinformatics Core, Thomas Jefferson University, Philadelphia, PA USA; 3 Institute for Biomedical Informatics, University of Pennsylvania, Philadelphia, PA USA

**Keywords:** Molecular biology, Environmental sciences

## Abstract

Early life lead (Pb) exposure is detrimental to neurobehavioral development. The quality of the environment can modify negative influences from Pb exposure, impacting the developmental trajectory following Pb exposure. Little is known about the molecular underpinnings in the brain of the interaction between Pb and the quality of the environment. We examined relationships between early life Pb exposure and living in an enriched versus a non-enriched postnatal environment on genome-wide transcription profiles in hippocampus CA1. RNA-seq identified differences in the transcriptome of enriched vs. non-enriched Pb-exposed animals. Most of the gene expression changes associated with Pb exposure were reversed by enrichment. This was also true for changes in upstream regulators, splicing events and long noncoding RNAs. Non-enriched rats also had memory impairments; enriched rats had no deficits. The results demonstrate that an enriched environment has a profound impact on behavior and the Pb-modified CA1 transcriptome. These findings show the potential for interactions between Pb exposure and the environment to result in significant transcriptional changes in the brain and, to the extent that this may occur in Pb-exposed children, could influence neuropsychological/educational outcomes, underscoring the importance for early intervention and environmental enrichment for Pb-exposed children.

## Introduction

A number of perinatal environmental and behavioral factors influence the development and maturation of the brain and subsequent cognitive and behavioral functioning. Early life exposure to environmental neurotoxicants such as lead (Pb) have the ability to derail neural and cognitive/behavioral development, with lifelong negative consequences. However, early developmental exposures to environmental neurotoxicants are inevitably intertwined with co- or sequentially occurring early life behavioral experiences that can vary widely in their character, and have the potential to modify the influences of environmental neurotoxicants on the brain and behavior. Thus, interactions between these influences have the potential to shape the brain and impact neurodevelopmental vulnerability or resilience, influencing the likelihood of behavioral/cognitive problems in childhood and later in adulthood.

Developmental Pb exposure is an ongoing public health concern in the United States and elsewhere. Adverse and persistent effects on cognition and behavior, including reductions in IQ and learning, memory, attention, and executive functioning deficits are associated with developmental Pb exposure in humans and in animal models (ex., Refs.^1–6^). Detrimental effects of Pb at blood levels (BLLs) below 10 µg/dL are well-documented and BLLs even below 5 µg/dL are associated with impaired cognition and school performance^7–10^, suggesting there may be no safe level of Pb in a child’s blood. Primary prevention of Pb exposure and the reduction or elimination of Pb from a child’s environment is critical but expensive, and sadly, prevention has focused primarily on attempting to reduce the exposure following identification of Pb-poisoned children, using children as proverbial canaries in the coal mine to identify toxic environments^11^. With wide-scale primary prevention lacking, the question becomes, what can be done to ameliorate the effects of Pb poisoning for the millions of children already exposed to Pb?

Socioeconomic status (SES) is one of the social/behavioral variables known to interact with Pb exposure. A child’s SES affects not only the likelihood of exposure to Pb but also the outcome from Pb exposure. Low SES children have a higher likelihood of being exposed to Pb, have higher BLLs, and more severe outcomes from exposure to Pb compared to high SES children^12^. Various concomitants of low SES may enhance Pb’s neurotoxicity^12^. Rutter^13^ hypothesized that economically disadvantaged children, because of a neuropsychological status rendered fragile by environmental influences, might be more vulnerable to the neurotoxic effects of Pb. Winneke and Kraemer^14^ reported that SES interacted with the effects of Pb on visual-motor integration and reaction time such that performance deficits were greater in lower SES Pb-exposed children than their higher SES counterparts. Similar effects were reported by Bellinger^12^. More recently, Marshall et al.^15^ reported that with increasing risk of Pb exposure, children from lower SES families had lower cognitive test scores, smaller cortical volumes, and smaller cortical surface areas compared with children from higher SES families. These authors suggested that reducing Pb-exposure risk might preferentially benefit low SES children, and that further understanding of the interacting factors of SES and Pb exposure will be critical for improving outcomes in children^15^.

We^16,17^ and others^18^ have shown that an animal analog of SES, i.e., environmental enrichment (EE) or impoverishment, can modify the effects of Pb on the brain and affect cognitive outcomes. We were the first to demonstrate that rearing rats in impoverished or enriched environments could modulate learning and memory in the Morris water maze (MWM) in Pb-exposed animals and affect trophic factor gene expression in the hippocampus^17^. This initial study demonstrated that Pb-induced neurotoxicity and cognitive deficits were potentially modifiable by environmental conditions, but it had several shortcomings in the use of only post-weaning Pb exposures, animals with relatively high BLLs (> 20 µg/dL), and use of behavioral extremes [enrichment vs. isolation (i.e., single animal housing)]. In a follow-up study, we examined effects of enriched environment (6 animals to a large enclosure containing various toys and climbing and nesting materials that were changed twice weekly) and non-enriched environment (animals housed 3 to a small enclosure containing no environmental enhancements) on Morris water maze (MWM) performance in males and females with different levels of perinatal Pb exposure (gestation through weaning). This study revealed complex interactions between sex, rearing environment, and Pb exposure, even at BLLs ≤ 10 µg/dL^16^. Other groups have also since described beneficial effects of environmental enrichment on spatial learning and memory and NR1 and BDNF gene expression in hippocampus in male rats with high levels of Pb exposure (1500 ppm from conception through weaning) and housing extremes of enrichment versus isolation rearing^18^. Others have also described effects of enrichment on MWM performance and long-term potentiation in the hippocampus in male rats with high perinatal Pb exposures (1500 ppm from conception through weaning)^19^.

Although the effects of early life Pb exposure, especially on hippocampal-based behaviors and expression of a few pre-selected genes, have been shown to be modifiable by the quality of the postnatal housing environment, the interactive effects of Pb exposure and quality of the environment on whole genome transcriptional profiles and functional networks are unknown. The present study sought to delineate the relationship between early life low level exposure to Pb and living in an enriched versus a non-enriched postnatal environment on gene transcription profiles in CA1 of the hippocampus, a part of the brain critically involved in learning and memory, using an RNA-sequencing (RNA-seq) approach, to provide new and detailed insight into the interactive effects of Pb exposure and the complexity of the postnatal environment at a molecular /systems level.

## Methods

### Animals and Pb exposure

**Figure 1:**
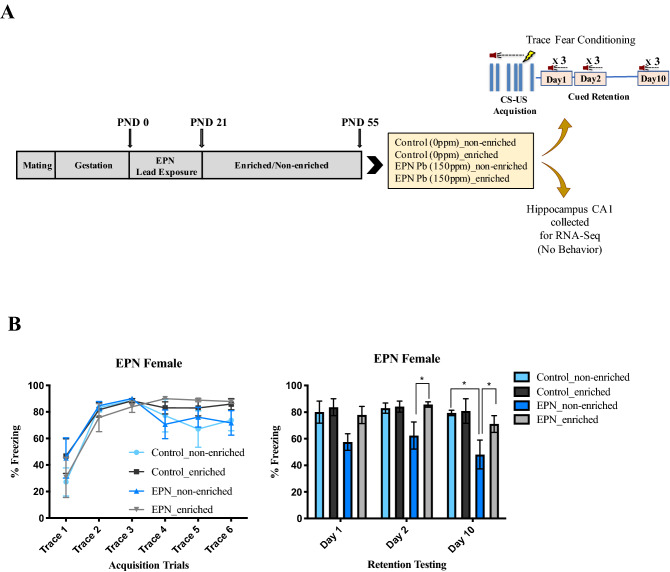
(**A**) Experimental study design. Long Evans rats received early postnatal (EPN) Pb exposure (150 ppm) via dams receiving Pb-containing diet beginning at postnatal day (PND) 1 through weaning (PND 21). On PND 21, female pups were randomly assigned to either the enriched or non-enriched environment. Controls (no Pb, 0 ppm) were similarly randomized to an enriched or non-enriched environment at weaning. The four experimental groups were: Control (0 ppm)_non-enriched, Control (0 ppm)_enriched, EPN Pb (150 ppm)_non-enriched, and EPN Pb (150 ppm)_enriched. At PND 55, some animals were randomly taken for trace fear conditioning while others were randomly selected for tissue collection. Behaviorally tested animals were assessed for post-conditioning memory retention at 1, 2 and 10 days after conditioning. Behaviorally naïve animals were euthanized to collect CA1 of the HIPP for RNA-sequencing. (**B**) Environmental enrichment mitigated negative effects of EPN Pb exposure on associative memory in the trace fear conditioning test. Lead-exposed animals living in the non-enriched environment had memory deficits detected at 1, 2 and 10 days after training. In contrast, Pb-exposed animals living in the enriched environment had no significant memory deficits. Data shown are group mean ± S.E.M; N = 6 per group; *p < 0.05.

The use of animals in this study was approved by the institutional animal care and use committee at Thomas Jefferson University and was in compliance with NIH Guidelines for the Care and Use of Laboratory Animals. The study was carried out in compliance with the ARRIVE guidelines. Animals received early postnatal (EPN) exposure to Pb. Dams were fed RMH 1000 chow with no added Pb during gestation and were fed chow with or without added Pb acetate (150 ppm) beginning at day of birth (postnatal day 1) and pups continued to receive this exposure to Pb through weaning at postnatal day 21. EPN Pb exposure at 150 ppm has been previously used in our studies and produces blood Pb values in offspring at postnatal day 21 of ~ 4.8 µg/dL^20^. Litters were culled to equal numbers of pups to standardize litter size at postnatal day 7, with an aim of having 8 pups per litter. Females with EPN Pb exposure were used for the present behavioral and molecular studies as previous research showed that EPN Pb exposure induces an associative memory deficit in females but not in males^21^. No more than one female from any litter was included in any experimental (housing) group. Post-weaning, rats were housed 3 to a standard cage (47.6 cm × 25.9 cm) with no added stimuli (non-enriched) or 6 to a cage (61 cm × 43.5 cm) containing a variety of toys, climbing and nesting materials, and tunnels that were changed twice per week (enriched group). Other than the differences already described, animals were handled in exactly the same manner and all animals were exposed to a 12 h:12 h light:dark cycle for the duration of the study. At postnatal day 55, some animals were used for behavioral testing and others were euthanized for collection of tissue for RNA sequencing. The experimental setup and timeline are shown in Fig. 1A.

### Trace fear conditioning

Trace fear conditioning was carried out as described by us previously^21^ using Ugo Basile Fear Conditioning chambers (30 cm deep × 34 cm wide × 41.5 cm high) and ANY-maze software (Version 4.99; Stoelting Co., Wood Dale, IL) to automatically measure freezing responses. The trace fear conditioning protocol consisted of habituation, conditioning, and short- and long-term retrieval testing at Days 1, 2 and 10 post conditioning as previously described^21^. Briefly, animals were habituated to the fear conditioning chamber, located within a dimly lit sound attenuating enclosure with white background noise, for 15 min one day prior to conditioning trials. During conditioning trials, animals were given 180 s to habituate to the test chamber and then given a series of 6 pairings of tone (conditioned stimulus; CS, 3000 Hz, 80 dB for 15 s) and shock (unconditioned stimulus; US, 0.8 mA for 1.0 s) with a 20 s trace period between CS and US, and pseudorandom intertrial intervals of 1–3 min. Freezing, defined by absence of all but respiratory movements, was measured every second during the 20 s of the trace period. Retention testing was performed at 1, 2 and 10 days post conditioning. For retention testing, animals were placed back into the same chamber in which they were initially trained but with different visual and olfactory cues. On each retention testing day, animals were habituated to the chamber for 180 s followed by presentation of 3 tones for 15 s each, in the absence of foot shock, with a pseudo random ITI (1–4 min.) between presentation of tones. Freezing was measured every second during the 20 s after tone presentation.

### Statistical analysis

Analysis of trace fear conditioning behavioral outcomes was performed by two-way ANOVA followed by posthoc pairwise comparisons using Prism 8.0, with a significance level set at P < 0.05 (Graphpad Software, San Diego, California USA).

### RNA sequencing

At postnatal day 55, naïve (no behavior) animals (N = 3 per group for RNA-seq) were euthanized by decapitation, brains removed fresh, sliced in a rat brain tissue matrix over ice, and CA1 of the hippocampus was quickly dissected from brain slices, frozen, and stored at – 80 °C until used. RNA was extracted manually using Qiagen RNeasy kits. Purity and RNA integrity were assessed using a NanoDrop 2000 spectrophotometer and an Agilent Bioanalyzer. Next generation sequencing libraries were prepared using the Illumina TruSeq Stranded Total RNA kit with ribosomal RNA depletion, following the manufacturer’s protocol. Total RNA libraries were sequenced at the Cancer Genomics and Bioinformatics Core facility, Thomas Jefferson University, using an Illumina NextSeq 500 and 75 bp paired-end chemistry.

### Sequencing analyses

Differentially expressed genes (DEGs) were identified through the in-house pipeline as follows. To remove adaptors and low-quality reads, read trimming was performed using trimmomatic (version: trimmomatic-0.38-1)^22^.Then raw paired-end FASTQ sequencing reads were mapped to the rat reference genome (rn6_refseq.gtf (rn6_refGene downloaded on Feb. 2017 from USSC Table browser)) using STAR (Spliced Transcripts Alignment to a Reference; version: STAR_2.6.1a_08-27) -alignMatesGapMax 2000 -outSAMtype BAM aligner^23^ followed by featureCounts ((version: 1.6.2) -T 5 -p -M -t exon -g gene_id)^24^ to calculate how many reads map to each gene/feature with parameters ‘-M’ (with multi-mapped reads) and ‘-t exon’ (within exon region). Detailed information of featureCounts can be found in the user guide, https://bioconductor.org/packages/release/bioc/vignettes/Rsubread/inst/doc/SubreadUsersGuide.pdf. Finally, DESeq2 [(version: 1.16.1) default arguments]^25^ identified DEGs by comparing the gene expression values of genes among the different groups of samples as (i) EPN_non-enriched vs Control_non-enriched, (ii) EPN_enriched vs EPN_non-enriched, (iii) Control_enriched vs Control_non-enriched, and (iv) EPN_enriched vs Ctrl_enriched. R-code for DESeq2 analysis can be found in Supplementary Material 9. Principal component analysis (PCA) and volcano plots were created by R package DESeq2 and ggplots^26^. GO and KEGG enrichment analysis was performed on DEGs above using clusterProfiler v3.16.0^27–29^.

Genes identified to have significant differential expression were further analyzed to define long non-coding RNA transcripts (lncRNA) and mitochondrial transcript subsets. lncRNA transcripts were identified based on Ensembl release 90 biotype definitions. To explore the potential interactions between differentially expressed (DE) lncRNAs and mRNAs identified in the RNA-seq datset, we constructed a lncRNA-mRNA co-expression network. The Pearson correlation coefficient of DE mRNA-lncRNA pairs was calculated to measure linear correlation and thus co-expression^30–33^. If the Pearson correlation coefficient was greater than 0.95, and the P value was less than 0.0001, we considered this DE lncRNA-mRNA pair to be linearly correlated and hence co-expressed. The co-expression network was established using Cytoscape software. Mitochondrial transcripts were identified to include both RNA transcribed from the mitochondrial genome as well as nuclear genes in the MitoMiner database known to contribute to mitochondrial function^34^. Pathway analyses were performed using Ingenuity Pathway Analysis (IPA; QIAGEN Inc.).

The Multivariate Analysis of Transcript Splicing tool (rMATs) was used to explore differential splicing events under the different experimental conditions^35^. In brief, rMATS identified differential alternative splicing (AS) events from two-group RNA-seq data with replicates (N = 3). rMATS is based on hierarchical framework that simultaneously models the variability among replicates and the estimation uncertainty of isoform proportion in each replicate. To estimate the p-value and FDR of the inclusion levels between two comparison groups of RNA-seq data, rMATS uses a likelihood-ratio test. In the present study, FDR < 0.05 was set as the threshold for differential AS events.

## Results

### Effects of Pb exposure and housing environment on associative memory function

Living in an enriched environment post-weaning modified EPN Pb-induced associative memory deficits (Fig. 1B). Animals housed in the non-enriched environment, Pb-exposed or not, tended to have less efficient learning compared with animals housed in the enriched environment, although these differences were not statistically significant. Pb-exposed animals from the enriched environment (EPN_enriched) had a learning profile indistinguishable from that of control, enriched animals (Control_enriched). EPN Pb-exposed animals raised in the non-enriched environment (EPN_non-enriched) had significant associative memory deficits detected at 2 and 10 days post-conditioning (Fig. 1B). There was a main effect of Pb (F (1, 15) = 13.81, p = 0.0021) and environment (F (1, 21) = 23.35, p < 0.0001) on memory. EPN_non-enriched animals had memory impairments compared to Control_non-enriched animals and the difference was significant at Day 10 (p = 0.03). EPN_enriched animals had no memory impairments and were not significantly different from Control_enriched animals (Fig. 1B). Significant differences in memory in EPN_enriched vs. EPN_non-enriched animals at Day 2 (p = 0.04) and Day 10 (p = 0.01) were observed (Fig. 1B).

### Overall influence of Pb exposure and housing environment on CA1 gene expression profiles

**Figure 2:**
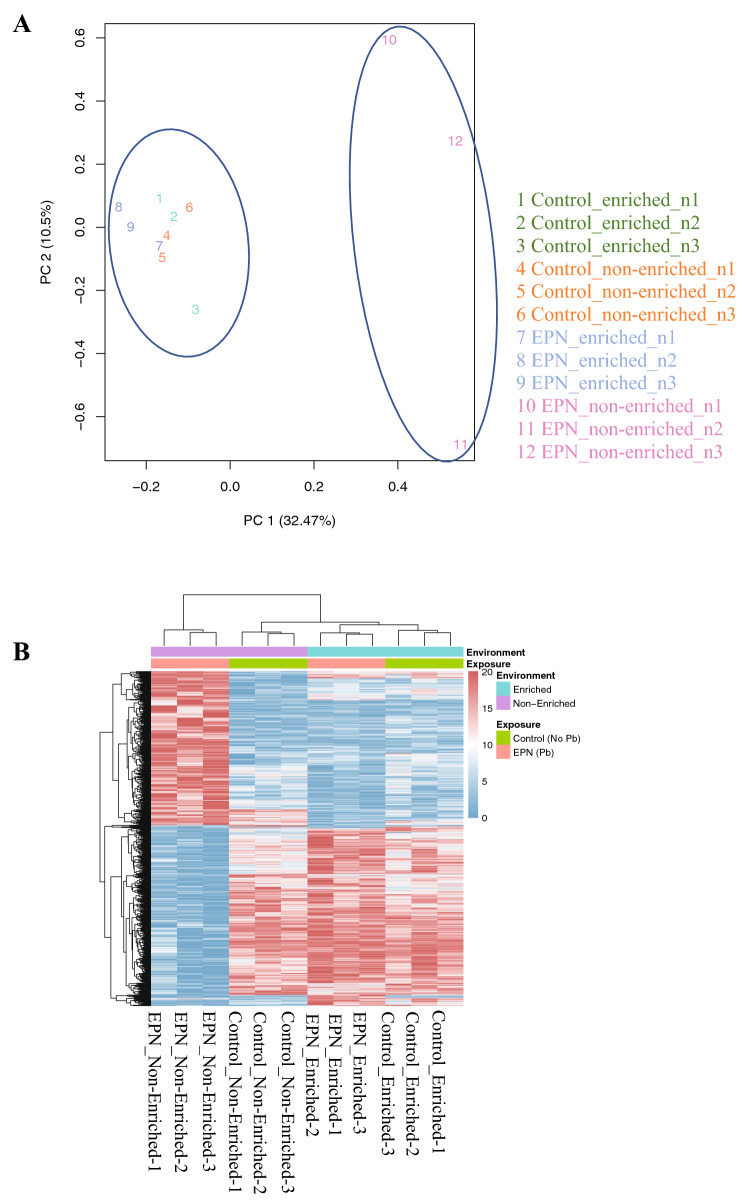
Principle component analysis (PCA) and hierarchical clustering of HIPP CA1 RNA-seq data. (**A**) PCA analysis across all samples and treatment conditions, based on overall gene expression profiles, shows EPN_non-enriched animals clustered in a distinct group compared to the other groups (Control_non-enriched, Control_enriched and EPN_enriched). (**B**) Heatmaps generated from unsupervised clustering of genes with the highest variation across the dataset show effects of Pb exposure and enrichment on gene expression patterns (FDR < 0.05, one-way ANOVA across all the treatment groups, see Supplementary Material 5). Transcriptomic changes in the EPN_non-enriched groups were distinct from those in the Control_(enriched or non-enriched) and EPN_enriched groups, while the heatmaps from EPN_enriched and Control_(enriched or non-enriched) animals were quite similar. N = 3 per group.

Early postnatal Pb exposure (150 ppm or 0 ppm) and housing environment (enriched or non-enriched) resulted into four experimental groups: Control (0 ppm)_non-enriched, Control (0 ppm)_enriched, EPN Pb (150 ppm)_non-enriched and EPN Pb (150 ppm)_enriched. Principal Component Analysis (PCA) across the samples and treatment conditions, based on overall gene expression profiles, was used to obtain an initial overview of the influences of Pb exposure and housing environment on the transcriptome (Fig. 2A). PCA identified two distinct clusters in the dataset. The samples from the EPN_non-enriched condition were grouped under one cluster while samples from the other three treatment conditions (EPN_enriched, Control_non-enriched and Control_enriched) were grouped into second cluster. The PCA result indicates that the post-weaning housing environment had no effect on the gene expression profiles of control (no Pb) animals and that the gene expression profile of Pb-exposed animals living in an enriched environment (EPN_enriched) was comparable to that of Control_non-enriched and Control_enriched animals. A similar result was evident in the heat map generated from one-way ANOVA data across all the treatment groups (FDR < 0.05), see Supplementary Material 5 (Fig. 2B). Although some differences can be observed in the transcriptional profiles between Control_enriched and Control_non-enriched groups, the most dramatic differences in transcriptional profiles was seen with the interaction of EPN Pb exposure and housing environment. The transcriptional profile of EPN_non-enriched animals is substantially different than that observed in EPN_enriched animals, whose profile is more similar to that observed in normal, non-Pb-exposed animals.

**Figure 3:**
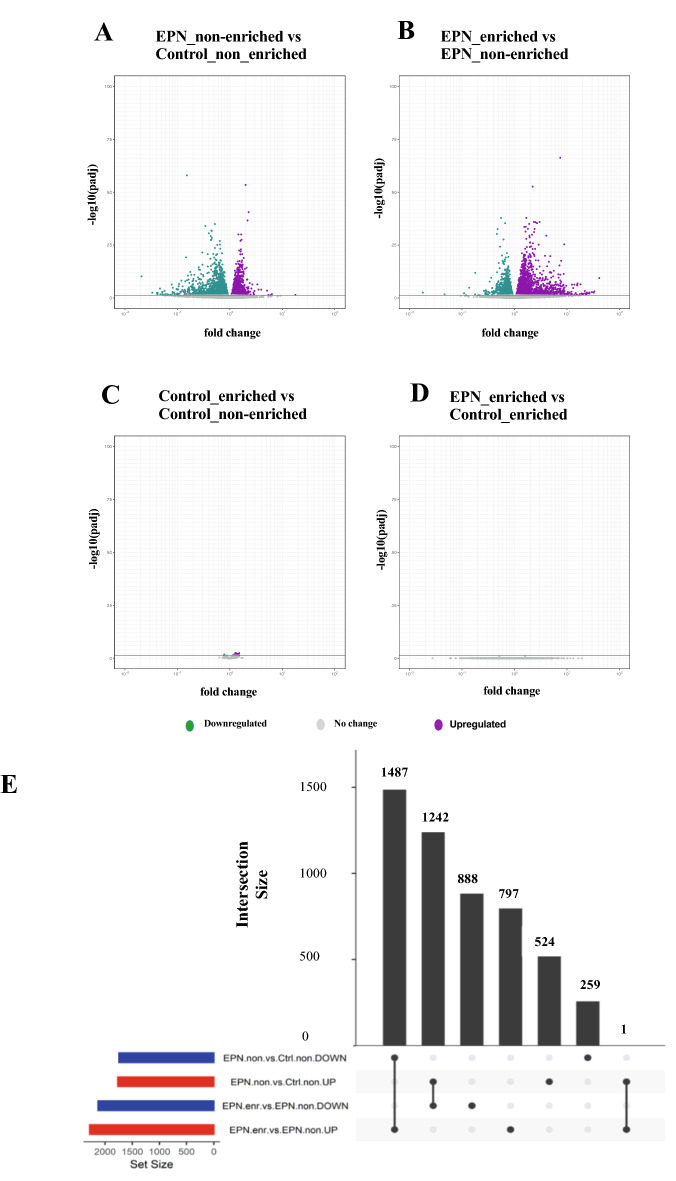
Analysis of differentially expressed genes (DEGs) showed that the Pb-altered transcriptome was further modified by environmental enrichment. (**A**–**D**) Volcano plots show log2 fold changes and associated p-values for DEGs in the various group comparisons: (**A**) EPN_non-enriched vs Control_non-enriched; (**B**) EPN_enriched vs EPN_non-enriched; (**C**) Control_enriched vs Control_non-enriched; (**D**) EPN_enriched vs Control_enriched. Green colored circles indicate genes that were significantly differentially downregulated; magenta-colored circles indicate genes that were significantly differentially upregulated (FDR < 0.05); grey colored circles indicate genes with expression unchanged. (**E**) Set comparison analysis of DEGs (FDR < 0.05) showed that environmental enrichment largely reversed Pb-induced transcriptome alterations. DEG sets are shown at the bottom with red and blue bars indicating the relative gene set size, where blue highlights downregulation and red highlights upregulation. Connectors joining two DEG set rows indicate overlap between those sets, where the bar heights on the graph represent set intersection sizes (noted by the numbers above each bar). Living in enriched environment resulted in upregulation of 1487 genes that were downregulated due to Pb exposure and downregulation of 1242 genes that were upregulated by Pb exposure.

Subsequent to the PCA analysis, a differential gene expression analysis (FDR < 0.05) was performed for each treatment group and revealed a total of 1746 downregulated and 1767 upregulated genes associated with Pb exposure only (EPN_non-enriched vs. Control_non-enriched). Living in the enriched environment further modified the Pb-altered transcriptome, resulting in 2130 downregulated and 2285 upregulated genes (EPN_enriched vs. EPN_non-enriched). A comparatively minimal effect of the enriched environment on the gene expression profile of control animals was observed, resulting in only 3 downregulated genes and 37 upregulated genes (Control_enriched vs. Control_non-enriched). Comparison of EPN_enriched vs. Control_enriched animals showed no differential expression of genes between the two conditions. Supplementary Table S1 lists the top 25 most significant genes (FDR < 0.05) in each group comparison (see Supplementary Material 2). Volcano plots (FDR < 0.05) depict expression changes of highly significant genes (Fig. 3A–D). Lead exposure resulted in significant downregulation of gene expression in non-enriched animals (Fig. 3A). Living in the enriched environment modified the Pb-altered transcriptome, resulting in a gene expression profile that was almost the mirror image of the Pb-altered profile in non-enriched animals (Fig. 3B). Minimal significant differential gene expression changes were observed as a consequence of living in the enriched environment in normal control animals and the transcriptome of EPN_enriched animals was indistinguishable from that of Control_enriched animals (Fig. 3C,D).

Based on the distinct transcriptome profile in Pb-exposed animals and the similarity in profiles between EPN_enriched and control animals (non-enriched or enriched), we further explored the extent to which living in the enriched environment was able to mitigate the Pb-induced gene expression changes (Fig. 3E). We first assessed the number of genes that were downregulated by Pb exposure but that might have been upregulated by environmental enrichment. Upon performing set comparison analysis (FDR < 0.05), we found 1487 genes that were downregulated due to Pb exposure in EPN_non-enriched animals were upregulated by living in the enriched environment in the EPN_enriched animals. We next investigated the extent to which the enriched environment was capable of reversing the expression of genes that were upregulated as a consequence of Pb exposure. We found a set of 1242 genes that were upregulated due to Pb exposure in EPN_non-enriched animals but became downregulated upon environmental enrichment in the EPN_enriched animals. The set comparison revealed 888 downregulated genes and 797 upregulated genes that were unique to only environmental enrichment effect (EPN_enriched vs EPN_non-enriched). We also found 524 upregulated and 259 downregulated genes due to Pb exposure (EPN_non-enriched vs Control_enriched) that were not reversed by living in the enriched environment. Thus, living in the enriched environment largely reversed Pb-induced alterations to the transcriptome. Supplementary material 6 lists the genes with expression modified by Pb exposure and with expression levels further modified by the enriched environment.

### Gene ontology (GO) analysis

**Figure 4:**
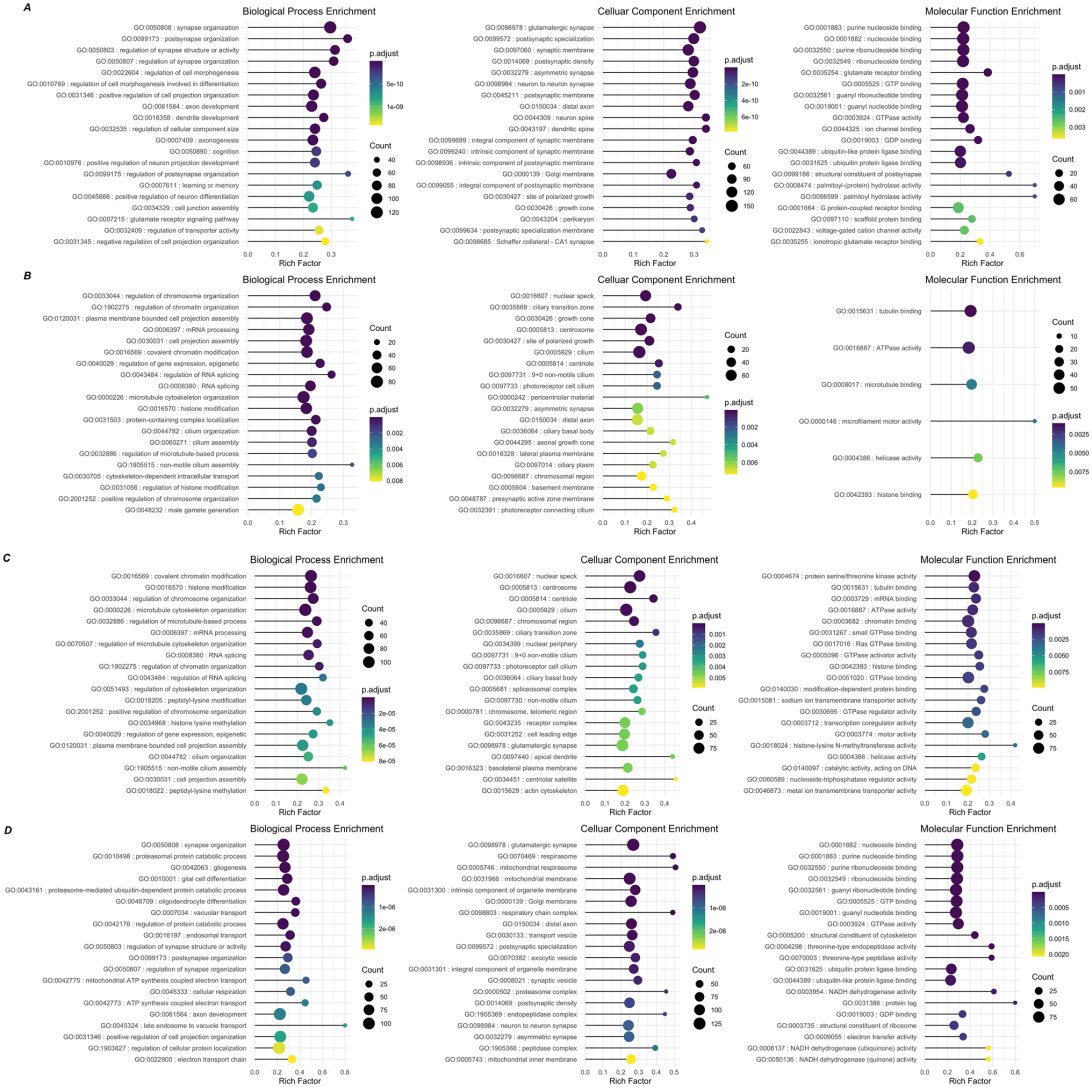
Gene ontology (GO) analysis of DEGs. Biological, Cellular, and Molecular processes that were significantly enriched due to Pb exposure associated with (**A**) upregulated or (**B**) downregulated genes (EPN_non-enriched vs Control_non-enriched). Panels (**C**) and (**D**) similarly show Biological, Cellular, and Molecular processes that were significantly enriched in response to environmental enrichment in Pb exposed animals (EPN_enriched vs EPN_non-enriched) and associated with upregulated or downregulated genes, respectively.

Gene Ontology (GO) analysis classified DEGs into three groups: biological processes, cellular components and molecular functions (Fig. 4). GO analysis was performed only on two comparisons, EPN_non-enriched vs Control_non-enriched and EPN_enriched vs EPN non-enriched. The other two comparisons, Control_enriched vs Control_non-enriched and EPN_enriched vs Control_enriched, had such a low number of DEGs that GO analyses could not be performed. Among the enriched GO processes, neuronal-related processes were among the top upregulated processes due to Pb exposure (Fig. 4A) and included processes such as synapse organization (GO:0050808), post-synapse organization (GO:0099173), regulation of synapse structure or activity (GO:0050803) and regulation of synapse organization (GO:0050807). The top downregulated processes associated with Pb exposure included regulation of chromosome organization (GO:0033044), regulation of chromatin organization (GO:1902275), plasma membrane bound cell projection assembly (GO:120031), mRNA processing (GO:0006397) and regulation of gene expression, epigenetic (GO:0040029) (Fig. 4B). Consistent with the results of the DEseq analysis, the biological, cellular, or molecular processes that were downregulated due to Pb exposure were upregulated by environmental enrichment and vice versa. For example, covalent chromatin modification (GO:0016569), histone modification (GO:0016570) and regulation of chromosome organization (GO:0033044) were among downregulated processes in the EPN_non-enriched group and were upregulated in the EPN-enriched group (Fig. 4C). We also performed Kyoto Encyclopedia of Genes and Genomes (KEGG) analysis on the DEGs dataset and the results of this analysis are shown in Supplementary Material 2, Supplementary Fig. S1). GO analysis was performed to gain insight into the biological significance of the genes with expression levels either reversed or not reversed by the environmental enrichment in the Pb-exposed animals (shown in Fig. 3E) and the results for this analysis are shown in the Supplementary Material 3. Among the genes with downregulated expression following Pb exposure that were reversed by upregulation in environmental enrichment, several RNA splicing biological process terms were present, with mRNA splice site selection (GO:0006376) having the highest enrichment.

### Ingenuity pathway analysis (IPA)

Ingenuity Pathway Analysis (IPA) was used to identify significant biological pathways (p < 0.05) that were most affected by the gene expression changes consequent to Pb exposure and the type of post-weaning housing environment. Table 1 shows the top 10 canonical pathways (activated and inhibited pathways ranked based on z-score ≥ 2 or ≤ − 2, respectively) identified as affected by Pb exposure and further modified by enriched environment. The activity pattern (z-score) for several canonical pathways altered by enriched environment in Pb-exposed animals was reversed when compared to non-enriched Pb-exposed animals (Table 1). The full list of significant biological pathways identified by IPA is shown in the Supplementary Table S2. This observation is similar to what was seen for DEGs where many gene expression changes induced by Pb exposure were reversed consequent to living in the enriched environment. IPA did not reveal any significant pathways altered in control animals housed in an enriched environment.

**Table 1:** Top canonical pathways from ingenuity pathway analysis (activation z-score ≥ 2 or ≤  − 2 and p-value < 0.05).

Canonical pathways	z-score
**EPN_non-enriched vs Control_non-enriched**	
Ephrin receptor signaling	4.1174614
Regulation of actin-based motility by rho	3.6565517
Ceramide signaling	3.57770876
Actin nucleation by ARP-WASP complex	3.57770876
Oxidative phosphorylation	3.5
RhoA signaling	3.18198052
Rac signaling	3.12358076
Signaling by rho family GTPases	2.94883912
Actin cytoskeleton signaling	2.82842712
fMLP signaling in neutrophils	2.69407953
Small cell lung cancer signaling	− 2.3094011
**EPN_enriched vs EPN_non-enriched**	
Small cell lung cancer signaling	2.49615088
Oxidative phosphorylation	− 5.7445626
Regulation of actin-based motility by rho	− 4.1576092
Superpathway of cholesterol biosynthesis	− 3.8729833
RhoA signaling	− 3.5688713
Ceramide signaling	− 3.544745
Actin nucleation by ARP-WASP complex	− 3.4112115
Ephrin receptor signaling	− 3.1013194
Cholesterol biosynthesis I	− 2.8284271
Cholesterol biosynthesis II (via 24,25-dihydrolanosterol)	− 2.8284271
Cholesterol biosynthesis III (via desmosterol)	− 2.8284271

Top 10 canonical pathways whose expression was modified by Pb exposure in animals from the non-enriched condition and in which enrichment reversed the direction of the gene expression change.

*EPN* early postnatal Pb exposure.

We also examined IPA results for the top diseases and disorders significantly associated with Pb exposure and environment. The top five significant diseases and disorders, molecular and cellular functions, and physiological system development and functions enriched for each group comparison are shown in the Supplementary Table S3. Notable findings were the enrichment of neurological disease, psychological disorders, cellular development, and nervous system development function in non-enriched Pb-exposed animals.

Using IPA Upstream Regulator Analysis, we also identified upstream regulators that may contribute to gene expression changes observed in our dataset (Table 2). In Pb-exposed animals from the non-enriched environment*,* JAK1/2 kinases were identified as top upstream regulators affected by Pb exposure, with a predicted activated state. We next examined the expression levels of the JAK1/2 signaling associated genes and found that Pb exposure resulted in upregulation of several genes (*BAIAP2, BCL11B, BHLHE22, C1QL3, CNIH2, CTXN1, DDN, HPCA, ICAM5, ITPKA, LHX2, NEUROD6, NPTX1, NRGN, PSD, PTK2B, RGS14, RPRML, RSPO2, SLC17A7, WIPF3; FDR* < *0.05*) and downregulation of two genes (*FAM81A* and *KCNG; FDR* < *0.05*). The expression change values are listed in the Supplementary Material 4. The next topmost upstream regulator with a predicted activation state was cAMP dependent protein kinase A, PKA. Transcription factors NFkB and CREB3 were also among top upstream regulators affected by Pb exposure but were not significantly upregulated. SHANK3, essential for proper functioning of synapses and a leading candidate gene for autism^36–38^, was also one of the top upstream regulators identified, although its directional state was not predicted by IPA. MAX, a transcription regulator that forms a complex with another transcription regulator MYC^39–42^ was significantly inhibited by Pb exposure.

**Table 2:** Top upstream regulators (ranked by z-score) affected by Pb exposure and housing condition.

Gene	p-value	Function	Activation z-score	Predicted activation state
**EPN_non-enriched vs Control_non-enriched**				
JAK1/2	6.90E−04	Kinase	3.962	Activated
Pka	2.18E−01	Enzyme complex	2.646	Activated
NFkB	3.30E−01	Transcription regulator complex	1.986	–
ERK	2.94E−01	Kinase	1.98	–
CREB3	6.63E−02	Transcription regulator	1.982	–
SHANK3	1.82E−04	Other	1.134	–
MAX	7.23E−03	Transcription regulator	− 2	Inhibited
MYC	3.62E−03	Transcription regulator	− 0.728	–
**EPN_enriched vs EPN_non-enriched**				
JAK1/2	1.33E−02	Kinase	− 3.545	Inhibited
SIRT2	5.65E−02	Transcription regulator	− 2.449	Inhibited
SOX10	1.78E−01	Transcription regulator	− 2	Inhibited
MTOR	5.22E−01	Kinase	− 2	Inhibited
SMAD3	1.80E−01	Transcription regulator	− 1.98	–
LEP	5.12E−01	Growth factor	− 1.8	–
SMYD1	8.90E−03	Transcription regulator	− 1.109	–
FSH	2.48E−02	Complex	− 1	–
SHANK3	3.52E−02	Other	− 0.447	–
HNF4A	7.69E−04	Transcription regulator	1.972	–
MYC	7.69E−04	Transcription regulator	0.847	–
**Control_enriched vs Control_non-enriched**				
PRKCA	4.78E−05	Kinase	–	–
MTA2	6.65E−03	Transcription regulator	–	–
NAB2	6.65E−03	Transcription regulator	–	–
Ppp1cc	6.65E−03	Phosphatase	–	–
CHD4	1.11E−02	Enzyme	–	–
EGR2	1.33E−02	Transcription regulator	–	–
IGBP1	1.33E−02	Phosphatase	–	–
Cdkn1c	2.85E−02	Other	–	–

Upstream regulators in red font are those in which the directional state was modified by Pb exposure in animals from the non-enriched condition and in which the directional state reversed direction in enriched animals. *EPN* early postnatal Pb exposure.

Compared to upstream regulators with mostly positive z-scores and predicted activated states in the Pb-non-enriched group, IPA analysis of upstream regulators in Pb-exposed enriched animals identified upstream regulators with mostly negative z-scores and with predicted inhibited states (Table 2). For example, JAK1/2 kinases, activated in association with Pb exposure, were inhibited in Pb-exposed animals housed in the enriched environment. Upon further examination we found that JAK1/2 signaling associated genes which were upregulated (mentioned above) due to Pb exposure in non-enriched animals (EPN_non-enriched vs Control_non-enriched) became downregulated in animals living in the enriched environment. Similarly, the two genes, *FAM81A* and *KCNG*, that were downregulated in Pb-exposed non-enriched animals, were upregulated in Pb-exposed animals housed in the enriched environment. Several upstream regulators were predicted to be inhibited in EPN_enriched vs. non-enriched: Sirtuin 2 (SIRT2), a histone deacetylase and abundantly found in brain regions like hippocampus, cortex and striatum was significantly inhibited^43,44^; SOX10, which plays a crucial role in glial differentiation was also inhibited^45^; mammalian target of rapamycin, MTOR, a serine/threonine kinase that plays an important role in neuronal growth and plasticity^46^. Other regulators that had negative z-score but were not predicted to be inhibitors included SMAD3, LEP, SMYD1, FSH and SHANK3. Two transcription regulators, HNF4A and MYC, had positive z-scores but their activation state was not predicated as an activator.

Eight genes were identified as upstream regulators in enriched control animals but none with an activation z-score or an identified activation state.

### Effects of Pb exposure and environment on mitochondrial gene expression

Our transcriptomic analysis revealed differential expression of several genes (FDR < 0.05; absolute FC ≥ 1.5) involved in mitochondrial metabolism and cellular energy regulation as a result of Pb exposure. Lead exposure induced a down regulation of important mitochondrial oxidative phosphorylation (OXPHOS)-related genes (Supplementary Table S4), including *NADH dehydrogenase 1 (ND-1)* and *NADH dehydrogenase 6 (ND-6)* that play crucial roles in the assembly of complex I, *Cytochrome B (Cytb),* the only mitochondrial encoded component of complex III, *Cytochrome C oxidase (COX) I, II* and *III*, electron receptors in the ATP production process, and *ATP synthase (ATP6)* of ATP synthase complex V, that produces ATP from ADP in the last step of the oxidative phosphorylation^47^. Negative effects of Pb exposure on mitochondrial gene expression were reversed by EE. Mitochondrial OXPHOS genes that were downregulated in EPN_non-enriched animals and upregulated in EPN_enriched animals are shown in Supplementary Table S5.

In addition to alterations in expression of mitochondrial OXPHOS genes, we also observed changes in gene expression (FDR < 0.05; absolute FC ≥ 1.5) for nuclear encoded mitochondrial genes (See Supplementary Material 2, Supplementary Table S6). As seen with the OXPHOS genes, environmental enrichment reversed the direction of expression changes in all of the genes significantly affected by Pb exposure, such that previously upregulated *Nat8l, P2ry1* and *Ucp2* genes were downregulated as a consequence of environmental enrichment and previously downregulated genes *Acadsb, Cpt1b, Cyp11b2, Ddit4, Dmgdh, Myo19, Slc9b2, and Slc8a3* genes were upregulated by environmental enrichment.

### Effects of Pb exposure and environment on alternative splicing (AS)

**Figure 5:**
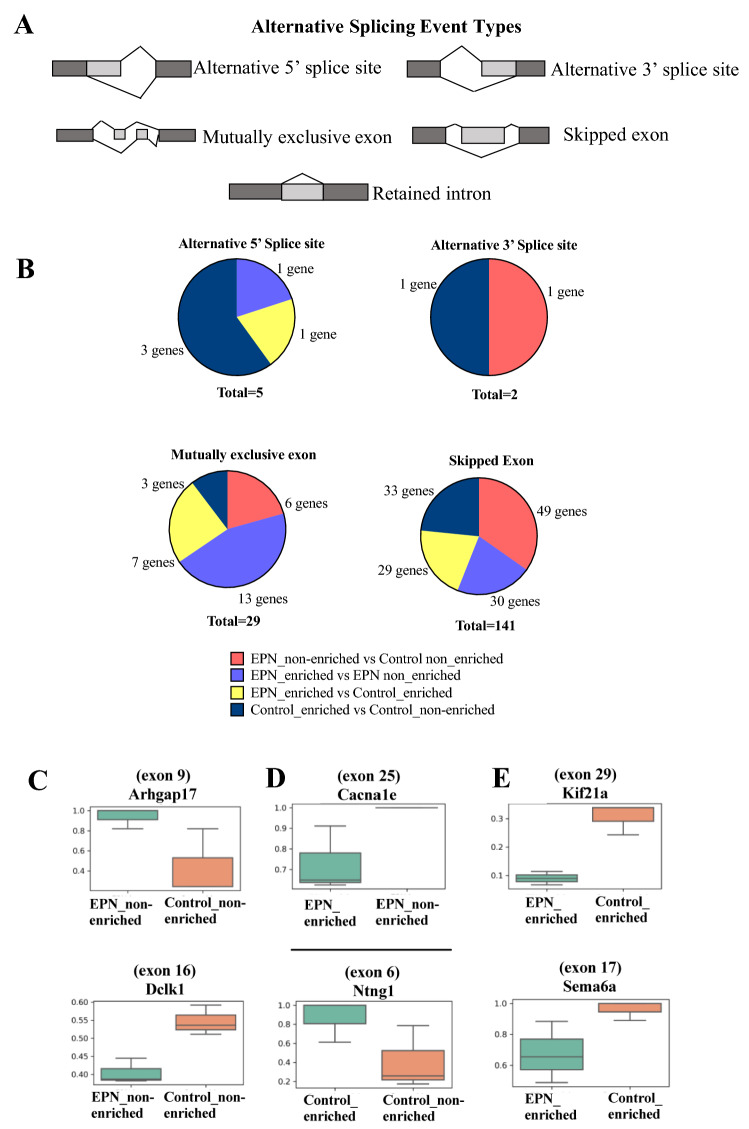
Alternative splicing (AS) events affected by Pb exposure and environmental enrichment. (**A**) Illustration of the five AS event types examined. (**B**) Types of AS events and number of involved genes detected in exposure-wise comparisons. The most significantly enriched AS event was skipped exon events. (**C**–**E**) Examples of differential exon skipping events in genes with neuronal functions in different exposure-wise comparisons. Boxplots showing the shifts in percent spliced in (PSI) values. Exon 9 in *Arhgap17* transcripts was significantly more included in the CA1 of Pb exposed, non-enriched animals compared to control, non-enriched animals. (**C**) Lead exposure resulted in a decrease in inclusion of exon 16 in *Dckl1* transcript as compared to non-enriched controls. (**D**) Exon 25 of Calcium Voltage-Gated Channel Subunit Alpha1E (*Cacna1e)* was significantly less included among the transcripts in EPN_enriched compared to EPN_non-enriched animals. In comparison to Control_non-enriched females, Exon 6 in Netrin G1 (*Ntng1*) transcripts were more included in Control_enriched females. (**E**) Exon 29 in Kinesin Family Member 21A (*Kif21a),* and exon 17 in Semaphorin 6A *(Sema6a)* transcripts were significantly less included among the transcripts in the CA1 of EPN_enriched animals vs Control_enriched animals for these two genes involved in brain development (FDR < 0.05 and delta PSI ≥ 0.05).

The enrichment of the GO terms RNA splicing (GO:0008380) and mRNA processing (GO:0006397) in the gene dataset led us to investigate the extent to which differential AS events, if any, occurred in association with Pb exposure and/or EE. The analysis focused on five basic types of AS events: alternative 5′ splice site, alternative 3′ splice site, mutually exclusive exon, skipped exon, and retained intron (Fig. 5A). Based on an FDR < 0.05, we identified skipped exon as the most abundant splicing event affected in Pb exposed non-enriched animals, Pb exposed enriched animals, and control enriched animals, suggesting that Pb exposure, environmental enrichment, and the combination of these factors may influence AS events (Fig. 5B). Further analysis (Fig. 5C–E) revealed skipped exon events for a number of genes associated with various neuronal functions, including *Rho GTPase Activating Protein 17(Arhgap17 or Nadrin)*, a GTPase-activating protein regulates calcium dependent exocytosis in nerve endings^48^, *Doublecortin-like kinase 1(Dclk1),* which plays important roles in neurogenesis and neural plasticity^49^, *Calcium Voltage-Gated Channel Subunit Alpha1E (Cacna1e)*, involved in neurotransmitter release^50,51^, and *Netrin G1 (Ntng1),* a cell adhesion molecule involved in regulating fear and anxiety behaviors^52^. Similar to what was observed in other analyses discussed earlier, we also observed environmental enrichment to reverse the direction of effect of Pb exposure on AS events (Table 3) that included the genes *Dckl1* (described above)*, cyclin dependent kinase like 1(Cdkl1)* a serine-threonine protein kinase^53–55^,* Cytochrome P450 Family 2 Subfamily R Member 1(Cyp2r1)* a member of the P450 superfamily of genes that are involved in maintaining the levels of neurochemicals in the brain^56^*, Influenza Virus NS1A Binding Protein (Ivns1abp) that* regulates pre-mRNA splicing^57–59^ and *Leukocyte Immunoglobulin Like Receptor B3a (Lilrb3a)* that is involved in immune response^60^*.* For example, the inclusion level of *Dckl1* alternatively spliced exon 16 that was decreased in EPN_non-enriched animals (Fig. 5C and Table 3) was higher in EPN_enriched animals.

**Table 3:** Environmental enrichment modifies Pb exposure-induced exon skipping events.

Gene symbol	Pb exposure regulated exon skipping events			Environmental enrichment modifies the pattern of Pb regulated exon skipping events		
	EPN_non-enriched PSI	Control_non-enriched PSI	EPN_non-enriched vs Control_non-enriched deltaPSI	EPN_enriched PSI	EPN_non-enriched PSI	EPN_enriched vs EPN_non-enriched deltaPSI
*Dclk1*	0.404	0.546	− 0.142	0.520	0.404	0.116
*Cdkl1*	0.993	0.88	0.113	0.888	0.993	− 0.105
*Cyp2r1*	0.927	0.588	0.339	0.530	0.927	− 0.397
*Ivns1abp*	0.056	0.144	− 0.088	0.496	0.056	0.109
*Lilrb3a*	0.848	0.254	0.594	0.193	0.848	− 0.656

*PSI* Percent Spliced In, *deltaPSI:* PSI (EPN_non-enriched—Control_non-enriched) or PSI (EPN_enriched—EPN_non-enriched), *EPN* early postnatal Pb exposure.

### Effects of Pb exposure and environment on long noncoding RNA (lncRNA) and mRNA networks

We detected a total of 69 differentially expressed lncRNAs in response to Pb exposure and all 69 lncRNAs were downregulated (FDR ≤ 0.05) (Supplementary Table S7). Expression of 63 of these 69 lncRNAs were upregulated in EPN_enriched vs. EPN_non-enriched animals. In control animals, environment (enriched or non-enriched) had no significant effect on expression of lncRNAs.

To explore the potential biological association between differentially expressed lncRNAs and mRNAs in our RNA-seq dataset, we constructed a lncRNA–mRNA co-expression network. A pair of lncRNA–mRNA was considered to be co-expressed if the expression levels of lncRNA and mRNA could be consistently correlated across the samples^30–33^. We identified more than 900 pairs in the network (See Supplementary Material 2, Supplementary Figs. S2 and S3). Among these correlations, we found 733 positive and 205 negative correlations in the EPN_non-enriched vs Control_non-enriched and 737 positive and 214 negative correlations in the EPN_enriched vs EPN_non-enriched. We found lncRNA AABR07065531.5 and LOC102550577 to be co-expressed with the highest number of mRNAs, 142 and 123, respectively, in EPN_non-enriched vs Control_non-enriched conditions and also in EPN_enriched vs EPN_non-enriched conditions (See Supplementary Material 2, Supplementary Figs. S4 and S5).

## Discussion

There are a number of different perinatal environmental and behavioral factors that may influence the development of the brain and affect subsequent cognitive and behavioral functioning. Early developmental exposure to an environmental neurotoxicant such as Pb, with adverse consequences for cognitive and behavioral development, is inevitably intertwined with co- or sequentially occurring early life experiences that can vary in their character. Both early exposures to an environmental risk factor such as Pb, and the nature of behavioral experiences, have the potential to modify the structure and function of the brain as well as the epigenetic and transcriptional landscape underlying physiological and plasticity-related processes. Dynamic interactions between these influences shape the brain and impact neurodevelopmental vulnerability or resilience, influencing the likelihood of behavioral/cognitive problems in childhood and later in adulthood.

The present study examined the impact of the complexity of the postnatal environment on behavioral and transcriptional outcomes related to early postnatal Pb exposure. We observed that living in an enriched environment had no effect on memory abilities of normal animals and caused minimal differences in gene expression profiles in CA1 in non-Pb-exposed animals raised in an enriched environment vs. animals raised in a non-enriched environment. In contrast, on the background of early life Pb exposure, the quality of the postnatal environment, non-enriched or enriched, had a significant impact on associative memory function and gene expression patterns in CA1. These findings are consistent with previous work from our lab^16,17^ and other groups^18^ that have described positive effects of environmental enrichment on cognitive/behavioral functions in Pb-exposed animals and positive effects on expression of select genes. The current work is the first to present a comprehensive picture of the interactions between early life Pb exposure and quality of the postnatal environment on the transcriptional landscape in hippocampus CA1 and the first to specifically assess these impacts on mitochondrial genes, lncRNAs, and AS events.

Principal component analysis (PCA) showed that both control groups and EPN_enriched animals grouped together while EPN_non-enriched animals were grouped separately. Heat maps showed very similar gene expression patterns for both control groups and EPN_enriched animals and these differed significantly from the gene expression pattern of the EPN_non-enriched animals. Additionally, living in the enriched environment was capable of reversing the expression changes of genes that were either downregulated or upregulated as a consequence of Pb exposure. Environmental enrichment was able to allow the brain to not only overcome the detrimental effects of early life Pb exposure on associative memory function but in many instances, to reverse the effects of Pb exposure on the CA1 transcriptome. These findings emphasize the importance of the quality of the postnatal environment for influencing the response of the brain to the detrimental effects of early life Pb exposure. Importantly, these findings indicate that the negative effects of early life Pb exposure on the brain and behavior are not immutable and can be influenced by the quality of the postnatal environment.

To the best of our knowledge, ours is the first exploration of the effects of Pb exposure and environment on the CA1 transcriptome and upstream regulators of gene expression. We show that several molecular pathways that were altered due to developmental Pb exposure showed changes in the opposite direction with enrichment. For example, Ephrin Receptor Signaling was among the top canonical pathways identified as differentially upregulated in the EPN_non-enriched group and downregulated in the EPN_enriched group. Ephrin (Eph) receptors and their ephrin ligands are present pre- and postsynaptically, and are involved in regulating synaptic transmission, plasticity, and memory formation^61^. Due to their complex and wide influence on other genes, Eph receptors are capable of influencing a variety of biological outcomes^62–64^. Dysregulated Eph receptor signaling has been implicated in abnormal neural development and altered synaptic transmission^65,66^. Gene networks of RhoA signaling, Rac signaling, and Signaling by Rho Family GTPases also play important roles in neuronal functioning, and were upregulated in the EPN_non-enriched group and downregulated in the EPN_enriched group. Interestingly, Eph receptors also regulate Rho GTPAse family genes including RhoA and Rac1 that are involved in synaptogenesis^64,67^.

One of the top upstream regulators affected by Pb exposure, JAK1/2 kinase, with a predicted activated state in EPN_non-enriched animals, was affected in the opposite direction (i.e., predicted inhibition state) in Pb-exposed, enriched animals. This is particularly interesting as in addition to being involved in brain inflammation and neuronal/glial survival^68,69^, JAK1/2 kinases and JAK-STAT signaling are also associated with hippocampal synaptic plasticity^68^ and may play roles in a variety of neurological disorders^68,70^.

Ingenuity Pathway Analysis identified top diseases and biological functions with transcriptomic changes in the different treatment groups, with results highly overlapping between the EPN_non-enriched and EPN_enriched groups. However, closer inspection of the data revealed that the direction of the changes in the genes constituting the top identified networks were in opposite directions in enriched versus non-enriched Pb-exposed animals and showed that environmental enrichment could reverse multiple gene pathway changes that occurred as a result of Pb exposure.

We believe that the current report is also the first exploratory study to identify effects of developmental Pb exposure and quality of housing environment on expression of mitochondrial genes. Mitochondria and the mitochondrial genome are known to be sensitive to Pb^71^. Chronic Pb exposure in humans also adversely affects mitochondrial calcium dependent enzyme systems, ATPases, and mitochondrial OXPHOS^72^. In the present study, mitochondrial genes encoding OXPHOS system components were significantly downregulated due to EPN Pb exposure in non-enriched animals, with the potential for compromising mitochondria ATP production. Environmental enrichment reversed the effects of Pb exposure on gene expression of these mitochondrial OXPHOS components. Several of the same mitochondrial genes affected in our study are also affected in humans occupationally exposed to Pb^71^. Interestingly, although EPN Pb exposure resulted in a significant reduction (log2FC > 1.5) in expression of OXPHOS pathway-related mitochondrial genes suggesting a downregulation of this pathway, IPA analysis suggested the OXPHOS pathway to be upregulated. The IPA-identified upregulated OXPHOS pathway included mainly 15 upregulated nuclear encoded OXPHOS mitochondrial genes with fold enrichments of small magnitudes (log2FC < 1.5). Gene expression changes of these magnitudes may not be biologically relevant at a single gene level but could become important if several genes with small expression changes are part of a single pathway. If mitochondria encoded OXPHOS genes were significantly downregulated at a high level but nuclear encoded OXPHOS genes showed small differences in expression and yet the OXPHOS pathway was identified as upregulated, what could be the significance of the observed changes in mitochondrial OXPHOS gene expression? One possibility is that there may be a differential effect of Pb exposure on the mitochondrial and nuclear genomes and net influences of mitochondria and nuclear (mitonuclear) crosstalk may determine the direction of change in the OXPHOS pathway^73–75^. Lead exposure was also associated with significant changes in gene expression for nuclear encoded mitochondrial genes [not associated with OXPHOS pathway and (log2FC ≥ 1.5)] and living in an enriched environment reversed the direction of expression change. These observations suggest that dysfunction of the OXPHOS pathway due to Pb exposure and its potential modification by environmental enrichment is worthy of further investigation.

Global AS alterations due to environmental exposures to chemicals, heavy metals, and stress have recently been reported^76–78^ and we identified perturbations in the normal pattern of AS in several genes due to Pb exposure. The most significantly affected AS event was exon skipping. Identification of a significantly abrupted splicing pattern in several genes suggests that Pb exposure may affect post-transcriptional regulatory mechanisms, however, the extent to which these Pb-affected alternative exons influence cellular functioning is not clear. A prior RNA-seq study showed an association between Pb exposure and splicing alterations in an in vitro model system, however very high Pb concentrations (30 µM) were used^79^. In our study, among the 49 genes showing an altered splicing pattern associated with Pb exposure, 5 genes showed an opposite pattern in Pb-exposed animals from the enriched environment. This suggests that the specific factors (cis or trans) perturbed by Pb exposure responsible for regulating the balance of normally spliced transcripts do not completely overlap with factors affected by environmental enrichment at a genome-wide level. Among the genes whose splicing pattern was abolished in the EPN_enriched group, *Dclk1* is interesting in that it plays an important role in synaptic plasticity and neurodevelopment^49,80,81^. Enriched environment also influenced the abundance of alternatively spliced mRNA isoforms in non-Pb-exposed animals, however, different genes were influenced by environmental enrichment in control animals versus Pb exposed animals. While the degree to which such changes have any physiological impact remains to be characterized, our findings suggest that AS may be an important aspect of the response of the brain to both Pb and environmental enrichment.

Another unique observation from our study is the differential expression of epigenetic regulators, lncRNAs, in response to Pb exposure and the ability of environmental enrichment to modify these Pb exposure-induced alterations in lncRNA expression. As the biological function of most of the lncRNAs is unknown, construction of lncRNA-mRNA networks allows us to predict the function of a lncRNA based on the function of mRNA co-expressed with a specific lncRNA^30–33,82^. We constructed co-expression networks between differentially expressed lncRNA and mRNAs in the EPN_non-enrich vs Control_non-enrich and EPN_enrich vs EPN_non-enrich revealing more than 900 relationships in each condition. Two lncRNAs, AABR07065531.5 and LOC102550577 were co-expressed with the greatest number of mRNAs. lncRNAs are known to interact with a variety of chromatin modifying proteins influencing the gene expression^83,84^ and we found that AABR07065531.5 is positively correlated with Lysine Demethylase 5A (*Kdm5a*) that encodes a chromatin regulator and regulates gene expression^85^. Previously, some studies also reported the involvement of lncRNAs in Pb-induced neuronal apoptosis^86–88^. Thus, in addition to studying expression of mRNAs, investigating the functional role of lncRNAs and their targets may provide an enhanced understanding of the molecular mechanisms involved in Pb-induced neurotoxicity.

The present study has a few limitations. First, this study used only females, so it is not known the extent to which the transcriptomic changes observed here are specific to females. We have previously reported sex-specific differences in gene expression changes due to developmental Pb exposure^89–91^ and therefore, it is quite possible that there may be a sex-dependent transcriptomic response to Pb ± environmental enrichment at a genome-wide level as well. Another limitation of our study is that we cannot differentiate between what may be adaptive or maladaptive responses from the experimental manipulations using only RNA-seq. Alterations in the transcriptome do not always reflect functional changes. Hence, future studies integrating various omics technologies (such as epigenomics, proteomics and metabolomics) with transcriptomics could potentially provide a more comprehensive understanding of the pathways and mechanisms involved in the response of the brain to Pb exposure and how environmental enrichment may modify those effects.

The present study did not detect an effect of enrichment on associative memory or transcriptional regulation in normal animals and there are several possible explanations for this. The fear conditioning paradigm used was designed to detect memory deficits in Pb-exposed animals and is less sensitive to detecting any possible enhancement above normal consequent to environmental enrichment due to a ceiling effect. Previously published studies investigating the effect of enrichment on gene expression in the brain (examining selected genes or gene expression using microarrays or RNA-seq) have reported significant changes in gene expression due to environmental enrichment^18,92,93^. The limited transcriptomic response detected in hippocampus CA1 in control animals in our study compared to previous studies could be related to differences in the duration of living in the enriched environment, type of enrichment, frequency with which objects in the environment were changed, numbers of conspecifics in the enrichment cage, and whether the group for comparison consisted of socially housed animals or isolation housed animals^94^, as well as brain region examined, species, strain, and sex. Different types of environmental enrichment (i.e., cognitive stimulation (exposure to toys), exercise (running wheel), motor learning) have been shown to have discrepant effects on memory in female mice, with exercise, but not cognitive stimulation, improving memory^95^. Our enriched environment did not include an exercise component (i.e., no running wheels). Similarly, studies in young adult rats that have demonstrated effects of environmental enrichment on learning and memory have been performed almost exclusively in males and our study was performed using females. This may be an important difference as sex-related in differences in response to enrichment have been reported^96^.

## Conclusions

In summary, our study shows widespread effects of developmental Pb exposure on the CA1 transcriptome and numerous biological processes, signaling pathways, nuclear and mitochondrial gene expression, AS events, and lncRNA expression that correspond with expression of associative memory deficits in EPN Pb-exposed rats. Enrichment provided post-weaning reversed many of Pb-induced transcriptional changes and improved associative memory functioning. It is increasingly appreciated that early life experiences affect broad life-course trajectories at least in part through modification of transcriptional regulation^97^. While the interactive effects of socioeconomic status (SES) and Pb exposure on neuropsychological outcomes in children have been known for quite some time, the effects of SES on the structure of the developing brain and the interaction with childhood Pb exposure has only recently begun to be explored^15^. In addition to the structural changes described by Marshall et al.^15^ the current findings suggest that interactions between Pb exposure and environment might also result in significant transcriptional changes in the brains of Pb-exposed children that could have profound influences on neuropsychological and educational outcomes, further underscoring the potential importance of early intervention and environmental enrichment especially for low SES, Pb-exposed children.

## Supplementary Information

Supplementary Information 1.

Supplementary Information 2.

Supplementary Information 3.

Supplementary Information 4.

Supplementary Information 5.

Supplementary Information 6.

Supplementary Information 7.

Supplementary Information 8.

Supplementary Information 9.

## Data Availability

Genomic data from this project is available in GSE180354.
