# Supplementary material for: Altered genome-wide hippocampal gene expression profiles following early life lead exposure and their potential for reversal by environmental enrichment
Source: Sci Rep. 2022 Jul 25;12:11937. doi: 10.1038/s41598-022-15861-9 (PMC9314447; doi:10.1038/s41598-022-15861-9)
Supplement: Supplementary file 1 — Supplementary Information 1. [file 41598_2022_15861_MOESM1_ESM.docx]

Altered Genome-Wide Hippocampal Gene Expression Profiles Following Early Life Lead Exposure and their Potential for Reversal by Environmental Enrichment

G. Singh^1*^, V. Singh^1^, T. Kim^3^, A. Ertel^2^, W. Fu^3^ and J.S. Schneider^1*^

Legends for Supplementary Information 3-9:

Supplementary Information 3: Gene Ontology analyses for the genes upregulated/downregulated by Pb exposure that were either modified by environmental enrichment or not.

Supplementary Information 4: The expression changes values of the JAK1/2 signaling associated genes from the RNA-seq dataset.

Supplementary Information 5: Result for ANOVA testing used to generate heatmap shown in Figure 2B.

Supplementary Information 6: List of DEGs identified in Figure 3E.

Supplementary Information 7: The inputs, read_counts.txt (read counts per genes) and sample_list.txt (list of samples with comparisons (CMPx)) for DESeq2.

Supplementary Information 8: The codes for the oneway ANOVA test code (onewayAnova.r) and the input file (normalized_by_DESeq.tsv: normalized read count per genes).

Supplementary Material 9: R code for DESeq2
