# Supplementary material for: Altered genome-wide hippocampal gene expression profiles following early life lead exposure and their potential for reversal by environmental enrichment
Source: Sci Rep. 2022 Jul 25;12:11937. doi: 10.1038/s41598-022-15861-9 (PMC9314447; doi:10.1038/s41598-022-15861-9)
Supplement: Supplementary file 2 — Supplementary Information 2. [file 41598_2022_15861_MOESM2_ESM.docx]

Altered Genome-Wide Hippocampal Gene Expression Profiles Following Early Life Lead Exposure and their Potential for Reversal by Environmental Enrichment

G. Singh^1*^, V. Singh^1^, T. Kim^3^, A. Ertel^2^, W. Fu^3^ and J.S. Schneider^1*^

**Table S1**. Top 25 genes differentially expressed (FDR <0.05) in EPN_non-enriched vs Control_non-enriched, EPN_enriched vs EPN_non-enriched, and Control_enriched vs Control_non-enriched. No differentially regulated genes were detected in EPN_enriched vs Control_enriched.

FC, fold change; FDR, False discovery rate.

| **EPN_non-enriched vs Control_non-enriched** | | |
| --- | --- | --- |
| Gene Symbol | Log2FC | FDR |
| *Ftx* | -2.7209788 | 1.07E-58 |
| *Nat8l* | 0.99905731 | 3.49E-54 |
| *Cx3cl1* | 1.19183611 | 2.76E-41 |
| *Shank3* | 1.12068646 | 2.22E-37 |
| *Cep290* | -0.9452038 | 1.10E-35 |
| *Kcnt1* | -1.5482919 | 9.52E-35 |
| *Clk1* | -1.1612988 | 1.25E-32 |
| *Luc7l3* | -1.1496686 | 1.97E-32 |
| *Pnisr* | -1.3347145 | 2.53E-31 |
| *Ptms* | 0.5414741 | 8.97E-31 |
| *Tril* | 0.72012849 | 8.97E-31 |
| *Ttc14* | -1.2091164 | 5.31E-30 |
| *Wsb1* | -1.1348109 | 3.78E-29 |
| *Mgat4b* | 0.74129779 | 2.81E-28 |
| *Clk4* | -1.1736063 | 2.81E-28 |
| *Gas5* | -1.2009668 | 4.01E-28 |
| *Adgrl1* | 0.66925585 | 9.58E-28 |
| *Macf1* | -0.6373695 | 1.12E-27 |
| *Prpf4b* | -0.6245139 | 5.65E-26 |
| *Alms1* | -0.7940547 | 7.70E-26 |
| *Lrp1b* | -0.9411576 | 2.25E-24 |
| *Golgb1* | -0.6089512 | 4.89E-24 |
| *Adgrb2* | 0.70421864 | 1.03E-23 |
| *Sbf1* | 0.59344799 | 3.39E-23 |
| *Ubqln2* | 0.68741607 | 1.04E-22 |
| **EPN_enriched vs EPN_non-enriched** | | |
| Gene Symbol | Log2FC | FDR |
| *Nat8l* | -0.8469866 | 1.47E-38 |
| *Ptms* | -0.5892905 | 4.39E-36 |
| *Cx3cl1* | -1.0656313 | 2.71E-33 |
| *Pcdh8* | -1.114982 | 5.50E-31 |
| *Tril* | -0.6912039 | 2.12E-28 |
| *Shank3* | -0.9109557 | 5.68E-25 |
| *Actg1* | -0.4820067 | 5.87E-22 |
| *Ssx2ip* | -0.7933141 | 7.86E-21 |
| *Mapk8ip1* | -0.3855103 | 3.64E-20 |
| *Sbf1* | -0.5471439 | 6.74E-20 |
| *Mgat4b* | -0.6055062 | 6.03E-19 |
| *Pmp22* | -0.7508515 | 2.51E-18 |
| *Adgrb2* | -0.6097169 | 5.13E-18 |
| *Abhd17c* | -0.7115071 | 8.34E-18 |
| *Mex3b* | -0.9941145 | 2.89E-17 |
| *Irf2bpl* | -0.7097407 | 4.37E-17 |
| *Pcdhgc3* | -0.6538774 | 9.56E-17 |
| *Eif4a1* | -0.3891804 | 8.94E-16 |
| *Guk1* | -0.3864781 | 3.26E-15 |
| *Plxnd1* | -0.9341141 | 7.54E-15 |
| *B4gat1* | -0.4455926 | 9.57E-15 |
| *Tmcc2* | -0.4613649 | 2.60E-14 |
| *Psd* | -0.5954633 | 3.11E-14 |
| *Ubl3* | -0.4620534 | 5.80E-14 |
| *Bsg* | -0.3614129 | 5.90E-14 |
| **Control_enriched vs Control_non-enriched** | | |
| Gene Symbol | Log2FC | FDR |
| *Id2* | -0.3531401 | 0.01679586 |
| *Id3* | -0.3443679 | 0.04105571 |
| *Pdzrn3* | -0.3823895 | 0.04355145 |

**Table S2.** Top Canonical Pathways from Ingenuity Pathway Analysis (Activation z-score ≥ 2 or ≤ ‑2 and p-value < 0.05)

| **EPN_non-enriched vs Control_non-enriched** | |
| --- | --- |
| **Canonical Pathways** | **z-score** |
| Ephrin Receptor Signaling | 4.1174614 |
| Regulation of Actin-based Motility by Rho | 3.6565517 |
| Ceramide Signaling | 3.57770876 |
| Actin Nucleation by ARP-WASP Complex | 3.57770876 |
| Oxidative Phosphorylation | 3.5 |
| RhoA Signaling | 3.18198052 |
| Rac Signaling | 3.12358076 |
| Signaling by Rho Family GTPases | 2.94883912 |
| Cardiac Hypertrophy Signaling | 2.88874152 |
| Actin Cytoskeleton Signaling | 2.82842712 |
| Cholecystokinin/Gastrin-mediated Signaling | 2.74397736 |
| fMLP Signaling in Neutrophils | 2.69407953 |
| Heparan Sulfate Biosynthesis | 2.67261242 |
| Wnt/Ca+ pathway | 2.67261242 |
| Unfolded protein response | 2.67261242 |
| Endocannabinoid Neuronal Synapse Pathway | 2.65495395 |
| G Beta Gamma Signaling | 2.65495395 |
| Phospholipase C Signaling | 2.5924757 |
| Leukocyte Extravasation Signaling | 2.53546276 |
| Cholesterol Biosynthesis I | 2.44948974 |
| Cholesterol Biosynthesis II (via 24,25-dihydrolanosterol) | 2.44948974 |
| Cholesterol Biosynthesis III (via Desmosterol) | 2.44948974 |
| Sphingosine-1-phosphate Signaling | 2.4140394 |
| GNRH Signaling | 2.40192231 |
| Chemokine Signaling | 2.40039679 |
| 3-phosphoinositide Biosynthesis | 2.3590713 |
| Superpathway of Cholesterol Biosynthesis | 2.33333333 |
| Heparan Sulfate Biosynthesis (Late Stages) | 2.30940108 |
| Remodeling of Epithelial Adherens Junctions | 2.30940108 |
| NRF2-mediated Oxidative Stress Response | 2.23606798 |
| 3-phosphoinositide Degradation | 2.21359436 |
| Ephrin B Signaling | 2.18282063 |
| Dermatan Sulfate Biosynthesis | 2.13808994 |
| Chondroitin Sulfate Biosynthesis | 2.13808994 |
| Cell Cycle: G2/M DNA Damage Checkpoint Regulation | 2.13808994 |
| Role of NFAT in Cardiac Hypertrophy | 2.13808994 |
| D-myo-inositol (1,4,5,6)-Tetrakisphosphate Biosynthesis | 2.13718683 |
| D-myo-inositol (3,4,5,6)-tetrakisphosphate Biosynthesis | 2.13718683 |
| FcŒ≥ Receptor-mediated Phagocytosis in Macrophages and Monocytes | 2.11695099 |
| p38 MAPK Signaling | 2.11057941 |
| Angiopoietin Signaling | 2.11057941 |
| Chondroitin Sulfate Biosynthesis (Late Stages) | 2.11057941 |
| Superpathway of Inositol Phosphate Compounds | 2.10042013 |
| D-myo-inositol-5-phosphate Metabolism | 2.081666 |
| PCP pathway | 2.0647416 |
| Reelin Signaling in Neurons | 2.0302589 |
| Xenobiotic Metabolism CAR Signaling Pathway | 2 |
| CDK5 Signaling | 2 |
|  |  |
| RhoGDI Signaling | -2.7947828 |
| Small Cell Lung Cancer Signaling | -2.3094011 |
| HIPPO signaling | -2.3094011 |
| FAT10 Cancer Signaling Pathway | -2.1213203 |
| Mitotic Roles of Polo-Like Kinase | -2 |
|  |  |
| **EPN_enriched vs EPN_non-enriched** |  |
| **Canonical Pathways** | **z-score** |
| Role of RIG1-like Receptors in Antiviral Innate Immunity | 2.64575131 |
| Role of BRCA1 in DNA Damage Response | 2.49615088 |
| Small Cell Lung Cancer Signaling | 2.49615088 |
| Amyotrophic Lateral Sclerosis Signaling | 2.33486893 |
| Apelin Pancreas Signaling Pathway | 2.33333333 |
|  |  |
| Oxidative Phosphorylation | -5.7445626 |
| Regulation of Actin-based Motility by Rho | -4.1576092 |
| Superpathway of Cholesterol Biosynthesis | -3.8729833 |
| EIF2 Signaling | -3.7811767 |
| RhoA Signaling | -3.5688713 |
| Ceramide Signaling | -3.544745 |
| Actin Nucleation by ARP-WASP Complex | -3.4112115 |
| Ephrin Receptor Signaling | -3.1013194 |
| UVC-Induced MAPK Signaling | -2.8867513 |
| Cholesterol Biosynthesis I | -2.8284271 |
| Cholesterol Biosynthesis II (via 24,25-dihydrolanosterol) | -2.8284271 |
| Cholesterol Biosynthesis III (via Desmosterol) | -2.8284271 |
| Cdc42 Signaling | -2.7456259 |
| Heparan Sulfate Biosynthesis | -2.6726124 |
| Rac Signaling | -2.6666667 |
| TCA Cycle II (Eukaryotic) | -2.6457513 |
| Superpathway of Geranylgeranyldiphosphate Biosynthesis I (via Mevalonate) | -2.6457513 |
| Remodeling of Epithelial Adherens Junctions | -2.4961509 |
| Signaling by Rho Family GTPases | -2.4797049 |
| Noradrenaline and Adrenaline Degradation | -2.4494897 |
| Mevalonate Pathway I | -2.4494897 |
| fMLP Signaling in Neutrophils | -2.3333333 |
| Dermatan Sulfate Biosynthesis | -2.32379 |
| Actin Cytoskeleton Signaling | -2.25 |
| Apelin Liver Signaling Pathway | -2.236068 |
| Ethanol Degradation II | -2.236068 |
| Chondroitin Sulfate Biosynthesis | -2.1380899 |
| Fatty Acid Œ≤-oxidation I) | -2.1213203 |
| Heparan Sulfate Biosynthesis (Late Stages) | -2.1105794 |
| 14-3-3-mediated Signaling | -2.0426487 |
| Dopamine Degradation | -2 |
| Ethanol Degradation IV | -2 |
| Glutathione Redox Reactions I | -2 |
| Tryptophan Degradation X (Mammalian, via Tryptamine) | -2 |
| Pentose Phosphate Pathway | -2 |
| Rapoport-Luebering Glycolytic Shunt | -2 |

EPN = early postnatal Pb exposure.

**Table S3.** Top Diseases and Biological Functions Results (with a p-value < 0.05) from Ingenuity Pathway Analysis

| **EPN_non-enriched vs Control_non-enriched** | | |
| --- | --- | --- |
| Diseases and Disorders | | |
| Name | p-value | # of molecules |
| Neurological Disease | 2.11E-02-1.29E-07 | 69 |
| Organismal Injury and Abnormalities | 2.11E-02-1.29E-07 | 158 |
| Cancer | 2.00E-02-1.08E-03 | 43 |
| Psychological Disorders | 2.11E-02-2.29E-03 | 11 |
| Skeletal and Muscular disorders | 1.49-02-2.50E-03 | 43 |
|  | | |
| Molecular and Cellular Functions | | |
| Name | p-value | # of molecules |
| Cellular Development | 2.06E-02-7.03E-27 | 557 |
| Cellular Growth and Proliferation | 2.06E-02-7.03E-27 | 489 |
| Cellular Assembly and Organization | 2.11E-02-1.05E-24 | 693 |
| Cellular Function and Maintenance | 2.00E-02-1.05E-24 | 775 |
| Cell Morphology | 2.00E-02-2.16E-19 | 465 |
|  | | |
| Physiological System Development and Function | | |
| Name | p-value | # of molecules |
| Nervous System Development and Function | 2.17E-02-7.03E-27 | 696 |
| Tissue Development | 2.17E-02-7.03E-27 | 665 |
| Organismal Development | 2.17E-02-8.41E-19 | 610 |
| Embryonic Development | 2.17E-02-1.92E-10 | 413 |
| Behavior | 2.17E-02-3.02E-10 | 165 |
|  | | |
| **EPN_enriched vs EPN_non-enriched** | | |
| Diseases and Disorders | | |
| Name | p-value | # of molecules |
| Neurological Disease | 2.97E-02-3.15E-04 | 75 |
| Organismal Injury and Abnormalities | 3.12E-02-3.15E-04 | 130 |
| Renal and Urological Disease | 1.83E-02-1.94E-03 | 25 |
| Cancer | 3.12E-02-2.65E-03 | 33 |
| Endocrine System Disorders | 1.34E-02-4.04E-03 | 35 |
|  | | |
| Molecular and Cellular Functions | | |
| Name | p-value | # of molecules |
| Cellular Assembly and Organization | 2.57E-02-2.06E-23 | 793 |
| Cellular Function and Maintenance | 3.16E-02-2.06E-23 | 911 |
| Cellular Development | 2.99E-02-4.35E-19 | 625 |
| Cellular Growth and Proliferation | 2.99E-02-4.35E-19 | 518 |
| Cell Morphology | 3.09E-02-1.79E-16 | 535 |
|  | | |
| Physiological System Development and Function | | |
| Name | p-value | # of molecules |
| Nervous System Development and Function | 3.16E-02-4.35E-19 | 795 |
| Tissue Development | 3.16E-02-4.35E-19 | 774 |
| Organismal Development | 2.97E-02-2.19E-14 | 699 |
| Embryonic Development | 2.97E-02-2.02E-08 | 506 |
| Tissue Morphology | 2.97E-02-1.81E-07 | 181 |
|  | | |
| **Control_enriched vs Control_non-enriched** | | |
| Molecular and Cellular Functions | | |
| Name | p-value | # of molecules |
| Cell cycle | 3.96E-03-2.71E-03 | 2 |
| Cellular development | 4.75E-02-3.28E-03 | 9 |
| Cellular growth and proliferation | 4.75E-02-3.28E-03 | 9 |
| Post-translational modification | 4.28E-02-5.39E-03 | 7 |
| Cellular function and maintenance | 4.75E-02-1.03E-02 | 6 |
|  | | |
| Physiological System Development and Function | | |
| Name | p-value | # of molecules |
| Behavior | 2.74E-02-1.94E-03 | 6 |
| Cardiovascular system development and function | 5.94E-03-3.28E-03 | 3 |
| Organ Development | 4.85E-02-3.28E-03 | 6 |
| Skeletal and Muscular System Development and Function | 3.32E-02-3.28E-03 | 3 |
| Tissue development | 4.85E-02-3.28E-03 | 8 |

EPN = early postnatal Pb exposure

**Table S4** Mitochondrial DNA nomenclature (gene names and symbols) for OXPHOS pathway genes.

| **mtDNA gene** | **Gene name** | **Gene Symbol** |
| --- | --- | --- |
| Complex I NADH dehydrogenase | NADH dehydrogenase 1  NADH dehydrogenase 2  NADH dehydrogenase 3  NADH dehydrogenase 4  NADH dehydrogenase 4L  NADH dehydrogenase 5  NADH dehydrogenase 6 | ND-1  ND-2  ND-3  ND-4  ND-4L  ND-5  ND-6 |
| Complex III cytochrome B | Cytochrome B | Cytb |
| Complex IV cytochrome C oxidase | Cytochrome C oxidase I  Cytochrome C oxidase II  Cytochrome C oxidase III | COX I  COX II  COX III |
| Complex V ATP synthase | ATP synthase 6  ATP synthase 8 | ATP-6  ATP-8 |

**Table S5.** Mitochondrial OXPHOS Genes with Expression Altered by Pb Exposure and Enrichment.

| **Mitochondrial genes** | **EPN_non-enriched vs Control_non-enriched (FDR ≤0.05, abs FC ≥ 1.5)** | **EPN_enriched vs EPN_non-enriched (FDR ≤0.05, abs FC ≥ 1.5)** |
| --- | --- | --- |
| *ND-1* | ↓ -1.2 | ↑ 0.70 |
| *ND-6* | ↓ -1.3 | ↑ 1.09 |
| *CYTB* | ↓ -0.53 | ↑ 0.62 |
| *COX I* | ↓ -0.79 | ↑ 0.71 |
| *COX II* | ↓ -0.76 | ↑ 0.49 |
| *COX III* | ↓ -0.66 | ↑ 0.40 |
| *ATP-6* | ↓-0.53 | ↑ 0.49 |

EPN = early postnatal Pb exposure

abs FC = absolute value of the fold change; FDR = False discovery rate; upward arrow = increased expression; downward arrow = decreased expression.

**Table S6.** Nuclear Encoded Mitochondrial Genes with Expression Altered by Pb Exposure and Environmental Enrichment.

| **Nuclear Encoded**  **Mitochondrial Genes** | **EPN_non-enriched vs Control_non-enriched (FDR ≤0.05, abs FC ≥ 1.5)** | **EPN_enriched vs EPN_non-enriched (FDR ≤0.05, abs FC ≥ 1.5)** |
| --- | --- | --- |
| *Nat8l* | ↑ 0.99 | ↓ -0.84 |
| *P2ry1* | ↑ 0.64 | ↓ -0.81 |
| *Ucp2* | ↑ 0.83 | ↓ -0.93 |
|  |  |  |
| *Acadsb* | ↓ -0.68 | ↑ 0.65 |
| *Cpt1b* | ↓ -0.92 | ↑ 1.27 |
| *Cyp11b2* | ↓ -3.27 | ↑ 3.11 |
| *Ddit4* | ↓ -0.60 | ↑ 0.75 |
| *Dmgdh* | ↓ -2.34 | ↑ 1.95 |
| *Myo19* | ↓ -0.86 | ↑ 1.04 |
| *Slc9b2* | ↓ -1.07 | ↑ 1.11 |
| *Slc8a3* | ↓ -0.78 | ↑ 0.58 |

EPN = early postnatal Pb exposure

abs FC = absolute value of the fold change; FDR = False discovery rate; upward arrow = increased expression; downward arrow = decreased expression.

**Table S7**. Environmental Enrichment Modifies Pb Exposure-Induced lncRNA Expression Changes

| **ENSEMBL** | **lncRNAs** | **Effect of Pb Exposure on lncRNA expression (log2FC)**  **(EPN_non-enriched vs Control_non-enriched)**  **FDR ≤0.05** | **Effect of enriched environment on Pb altered lncRNA expression (log2FC)**  **(EPN_enriched vs EPN_non-enriched)**  **FDR ≤0.05** |
| --- | --- | --- | --- |
| ENSRNOG00000051257 | Rn50_X_0752.3 | -1.72255166 | - |
| ENSRNOG00000051325 | Rn50_X_0667.2 | -3.712215812 | - |
| ENSRNOG00000051384 | AABR07002337.1 | -0.980605344 | 1.031231673 |
| ENSRNOG00000051572 | LOC102555189 | -1.981171004 | 2.058155886 |
| ENSRNOG00000051588 | AABR07024669.1 | -3.497364038 | 3.289501289 |
| ENSRNOG00000051725 | AABR07048878.1 | -2.254363235 | 2.23314275 |
| ENSRNOG00000051731 | AABR07059004.1 | -0.983446097 | 1.444551861 |
| ENSRNOG00000052018 | AABR07052441.1 | -2.391042728 | 2.532730827 |
| ENSRNOG00000052421 | AABR07012475.2 | -1.355357066 | 1.700556415 |
| ENSRNOG00000052632 | AABR07034293.1 | -5.036580828 | 5.995244256 |
| ENSRNOG00000053005 | AABR07030890.1 | -2.705055709 | 3.005209559 |
| ENSRNOG00000053185 | AABR07006889.1 | -1.747256209 | 2.182970935 |
| ENSRNOG00000053206 | LOC688442 | -2.122394867 | 2.342757189 |
| ENSRNOG00000053708 | AABR07063613.1 | -1.963722623 | 2.193030718 |
| ENSRNOG00000053722 | AABR07069218.2 | -2.958676543 | 3.005208805 |
| ENSRNOG00000053849 | AABR07064099.1 | -3.844989187 | - |
| ENSRNOG00000054632 | AABR07051882.1 | -0.914746176 | 0.967945109 |
| ENSRNOG00000054768 | AABR07050487.1 | -2.62737525 | 2.888859879 |
| ENSRNOG00000054849 | AABR07031388.1 | -1.46172004 | 1.926562728 |
| ENSRNOG00000054984 | AABR07026483.1 | -0.881205978 | 1.255984655 |
| ENSRNOG00000054990 | AABR07060133.1 | -1.258772892 | 1.572463757 |
| ENSRNOG00000055302 | AABR07051326.1 | -1.2205156 | 1.322724707 |
| ENSRNOG00000055317 | AABR07028908.2 | -0.87962503 | 1.16417237 |
| ENSRNOG00000055687 | AABR07035273.1 | -1.06870942 | 1.34206293 |
| ENSRNOG00000055838 | AABR07025263.1 | -1.863170944 | 1.84567487 |
| ENSRNOG00000055850 | LOC100910750 | -1.80935768 | 2.300508744 |
| ENSRNOG00000056410 | AABR07033184.1 | -2.099642733 | 2.295946797 |
| ENSRNOG00000057137 | AABR07005752.1 | -0.738549476 | 1.105683072 |
| ENSRNOG00000057243 | AABR07071228.1 | -2.122387722 | 1.977941124 |
| ENSRNOG00000057840 | AABR07026473.1 | -1.579387571 | 1.657458823 |
| ENSRNOG00000058115 | AABR07041709.1 | -1.664488361 | 1.790911765 |
| ENSRNOG00000058323 | LOC102550026 | 1.042872766 | - |
| ENSRNOG00000058335 | AABR07051879.1 | -1.129562644 | 1.18389323 |
| ENSRNOG00000058425 | AABR07000385.2 | -1.997371123 | 2.7998292 |
| ENSRNOG00000058526 | AABR07033234.1 | -2.951413671 | 2.765380997 |
| ENSRNOG00000059087 | LOC102546889 | -0.995927959 | 1.403306406 |
| ENSRNOG00000059262 | AABR07024786.1 | -2.304965586 | 2.574953055 |
| ENSRNOG00000059271 | AABR07058985.2 | -0.92367235 | 1.243483763 |
| **ENSEMBL** | **lncRNAs** | **Effect of Pb Exposure on lncRNA expression (log2FC)**  **(EPN_non-enriched vs Control_non-enriched)**  **FDR ≤0.05** | **Effect of enriched environment on Pb altered lncRNA expression (log2FC)**  **(EPN_enriched vs EPN_non-enriched)**  **FDR ≤0.05** |
| ENSRNOG00000059374 | AABR07024457.1 | -1.833167296 | 1.923434204 |
| ENSRNOG00000059660 | AABR07065531.5 | -1.495151425 | 1.777065848 |
| ENSRNOG00000059710 | AABR07039236.1 | -1.891240077 | 1.864567404 |
| ENSRNOG00000059761 | AABR07007798.1 | -2.490426192 | 2.600253012 |
| ENSRNOG00000059784 | AABR07071697.1 | -1.356341474 | 1.525603014 |
| ENSRNOG00000059825 | Rn50_20_0046.5 | -2.243452418 | 2.62616845 |
| ENSRNOG00000059974 | AABR07053283.1 | -2.533908155 | 2.795245465 |
| ENSRNOG00000060009 | AABR07005794.1 | -2.050900998 | 1.977099148 |
| ENSRNOG00000060195 | AABR07051882.2 | -0.971025389 | 0.975846895 |
| ENSRNOG00000060305 | AABR07008462.1 | -2.72090895 | 2.600714228 |
| ENSRNOG00000060509 | LOC102556339 | -2.651723353 | 3.625547507 |
| ENSRNOG00000060635 | AABR07052897.3 | -1.680654227 | - |
| ENSRNOG00000060675 | AABR07055801.1 | -1.752429092 | 1.750037992 |
| ENSRNOG00000060937 | AABR07059258.1 | -1.207469798 | 1.282969854 |
| ENSRNOG00000060961 | AABR07070246.1 | -2.178580818 | 2.677627682 |
| ENSRNOG00000060974 | AABR07065448.1 | -1.887583424 | 2.01703774 |
| ENSRNOG00000060990 | AABR07000382.1 | -1.974505679 | 1.857763665 |
| ENSRNOG00000061194 | AABR07003751.1 | -2.515865361 | - |
| ENSRNOG00000061467 | AABR07043772.1 | -1.504218585 | 1.656950267 |
| ENSRNOG00000061472 | AABR07033720.1 | -0.760862599 | 0.948425584 |
| ENSRNOG00000061539 | LOC102550577 | -1.873122449 | 2.248857512 |
| ENSRNOG00000061691 | LOC102550455 | -1.470170571 | 1.621612606 |
| ENSRNOG00000061869 | AABR07035012.1 | -2.559936958 | 3.122192971 |
| ENSRNOG00000061917 | AABR07042840.1 | -1.422975571 | 1.696672231 |
| ENSRNOG00000061918 | AABR07069008.3 | -2.862966144 | 3.239972894 |
| ENSRNOG00000061944 | AABR07060473.1 | -1.909800688 | 1.950258092 |
| ENSRNOG00000062076 | AABR07061237.1 | -2.642140044 | 2.882305127 |
| ENSRNOG00000062127 | Rn60_1_2212.2 | -1.685454841 | 1.96304514 |
| ENSRNOG00000062155 | Rn60_1_2212.3 | -1.691111577 | 1.834783409 |
| ENSRNOG00000062158 | Rn60_1_2212.4 | -1.638946585 | 1.927982255 |
| ENSRNOG00000062170 | Pvt1 | -0.739623894 | 0.825975395 |

EPN = early postnatal Pb exposure

FC = fold change; FDR = False discovery rate; negative number signifies downregulation; positive number upregulation

**B**

**A**

**D**

**C**

Downregulated genes genes

Upregulated genes

**Figure S1**. Kyoto Encyclopedia of Genes and Genomes (KEGG) enrichment scatter plots of upregulated and downregulated DEGs in EPN_non-enriched vs Control_non-enriched (A and B, respectively) and EPN_enriched vs EPN_non-enriched (C and D, respectively). The y-axis represents the pathway name, and the x-axis represents the Rich factor. The color indicates the q-value and the dot size corresponds to the number of different genes affected. KEGG analysis revealed enrichment of genes associated with glutamatergic and dopaminergic synapses, and various genes associated with neurodegenerative diseases such as Huntington’s, Alzheimer’s, and Parkinson’s diseases and Amyotrophic lateral sclerosis that were upregulated in EPN_non-enriched animals (A) and downregulated in Pb-exposed, enriched animals (D).


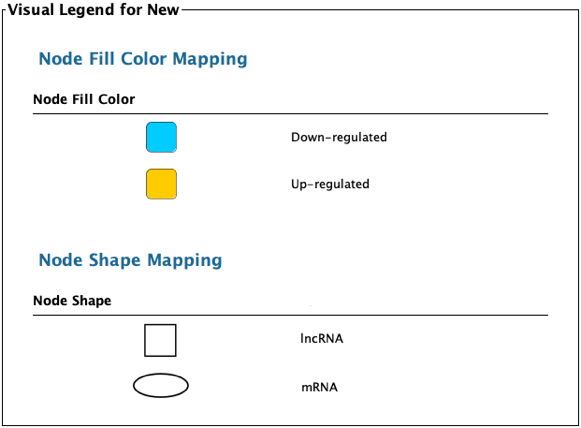


**Figure S2**. Co-expression network between differentially expressed lncRNAs and mRNAs in the EPN_non-enriched vs Control_non_enriched. 733 positive and 205 negative correlations were detected between differentially expressed lncRNAs and mRNAs. Solid lines indicate positive correlations, and dashed lines indicate negative correlations. (|PCC| >0.95, P<0.0001)

PCC, Pearson Correlation Coefficient

**
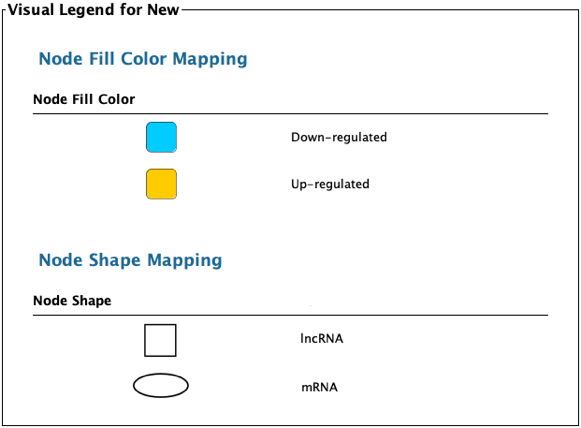
Figure S3**. Co-expression network between differentially expressed lncRNAs and mRNAs in the EPN_enriched vs EPN_non_enriched. 737 positive and 214 negative correlations were detected between differentially expressed lncRNAs and mRNAs. Solid lines indicate positive correlations, and dashed lines indicate negative correlations. (|PCC| >0.95, P<0.0001)

PCC, Pearson Correlation Coefficient

A.


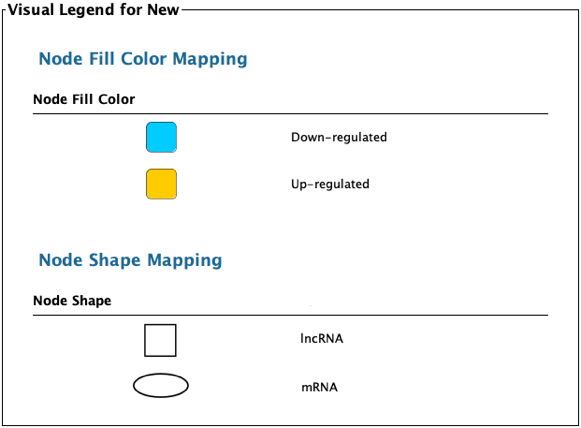

B.

**Figure S4**. Co-expression network between differentially expressed lncRNA AABR07065531.5 and mRNAs in the A) EPN_non-enriched vs Control_non_enriched and B) EPN_enriched vs EPN_non_enriched groups. 111 positive correlations and 31 negative correlations were found in EPN_non-enriched vs Control_non_enriched and 110 positive correlations and 32 negative correlations were found in EPN_enriched vs EPN_non_enriched. (|PCC| >0.95, P<0.0001)

PCC, Pearson Correlation Coefficient

A.


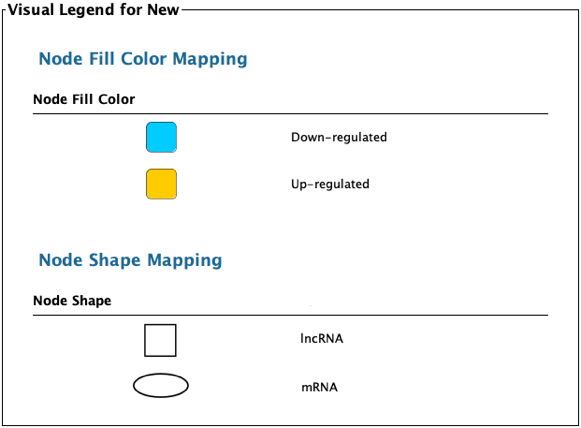

B.

**Figure S5**. Co-expression network between differentially expressed lncRNA LOC102550577 and mRNAs in the A) EPN_non-enriched vs Control_non_enriched and B) EPN_enriched vs EPN_non_enriched groups. 93 positive correlations and 30 negative correlations were found in each condition. (|PCC| >0.95, P<0.0001)

PCC, Pearson Correlation Coefficient
