# Supplementary figures and images for: Altered genome-wide hippocampal gene expression profiles following early life lead exposure and their potential for reversal by environmental enrichment
Source: Sci Rep. 2022 Jul 25;12:11937. doi: 10.1038/s41598-022-15861-9 (PMC9314447; doi:10.1038/s41598-022-15861-9)

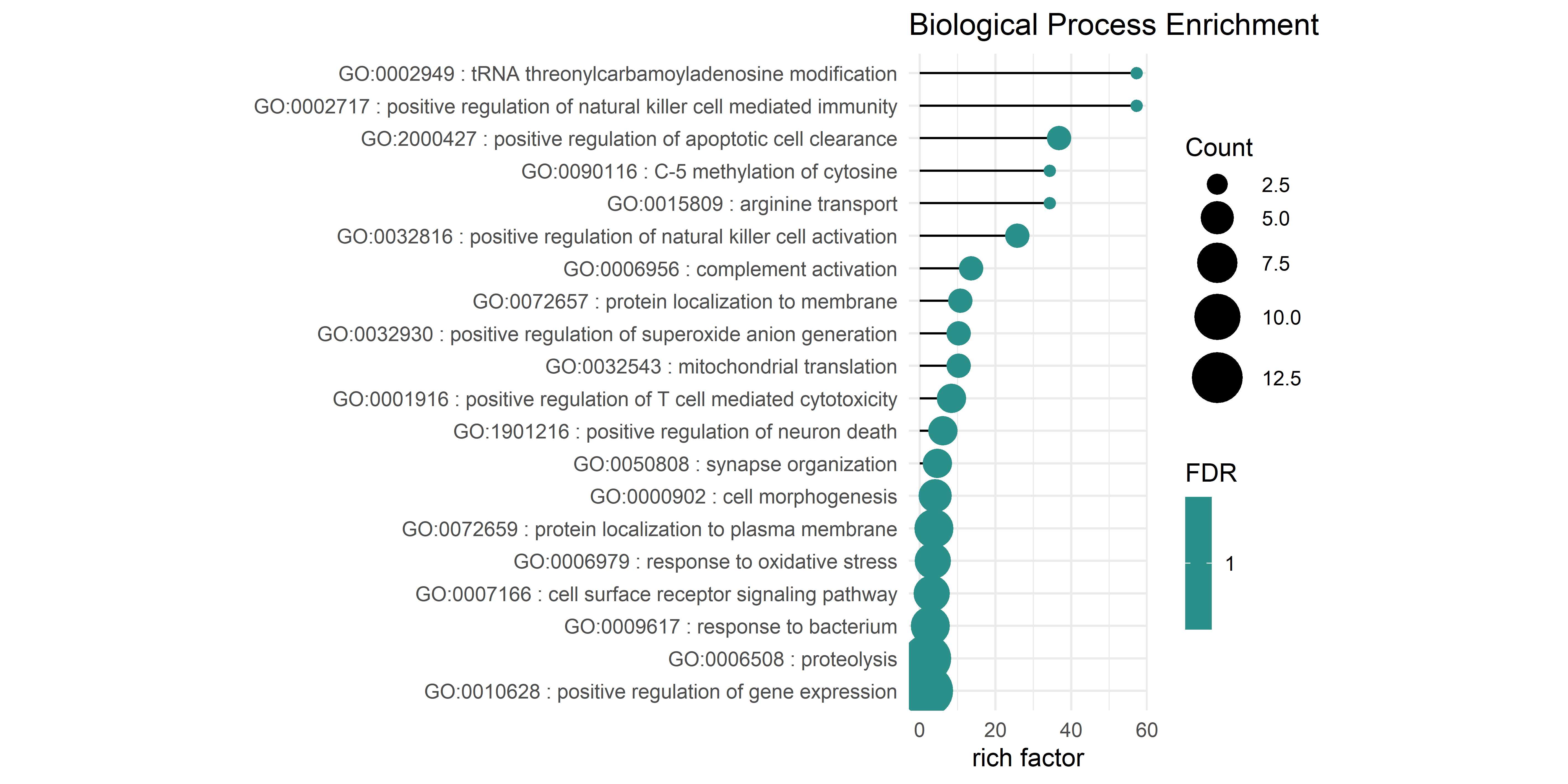

Supplement: Supplementary file 3 — Supplementary Information 3. [file 41598_2022_15861_MOESM3_ESM.zip › Singh et al_Supp Material_3_GO_analysis_Fig3/EPN.DownAndSustained.GOBP.png]

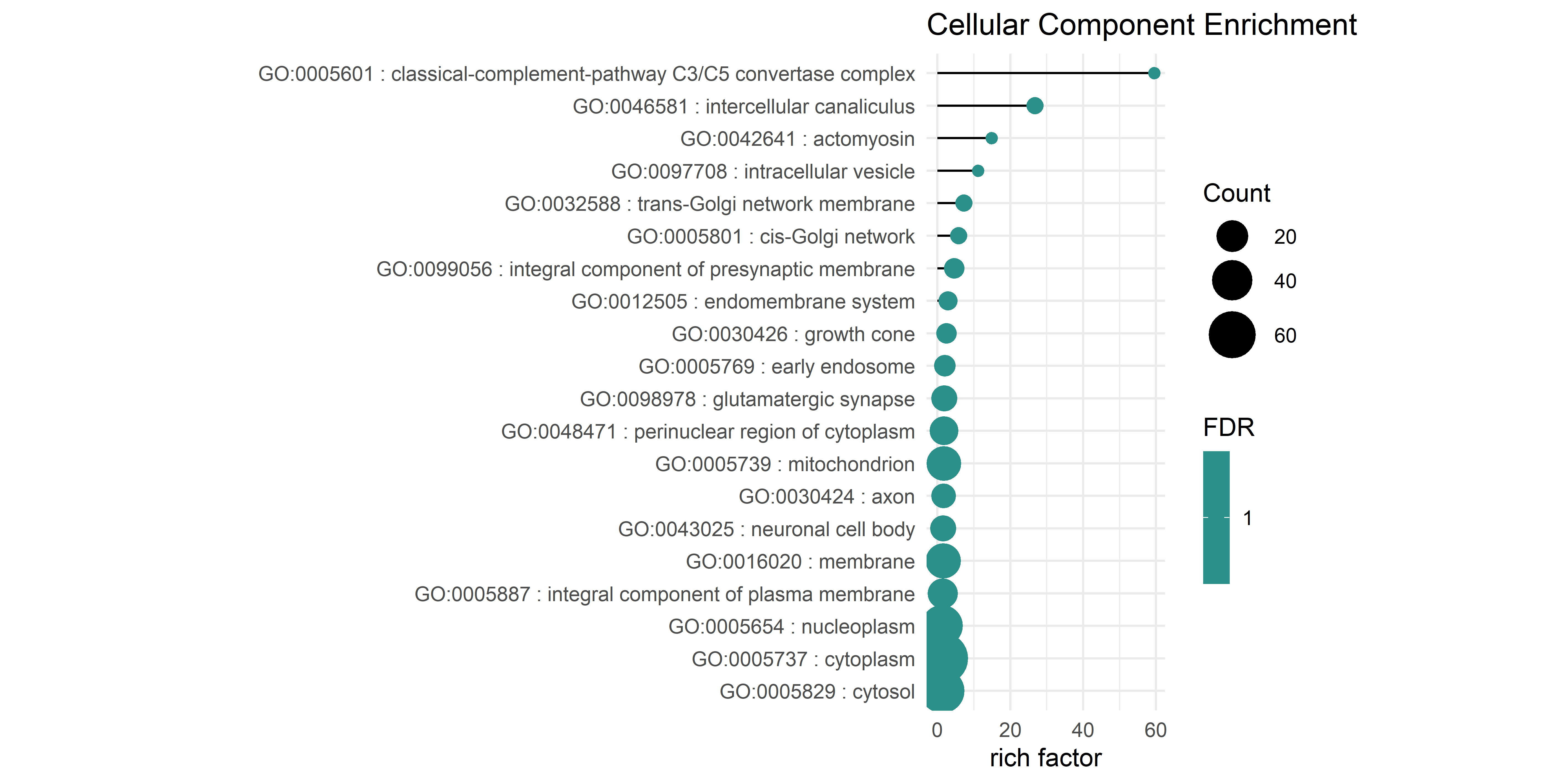

Supplement: Supplementary file 3 — Supplementary Information 3. [file 41598_2022_15861_MOESM3_ESM.zip › Singh et al_Supp Material_3_GO_analysis_Fig3/EPN.DownAndSustained.GOCC.png]

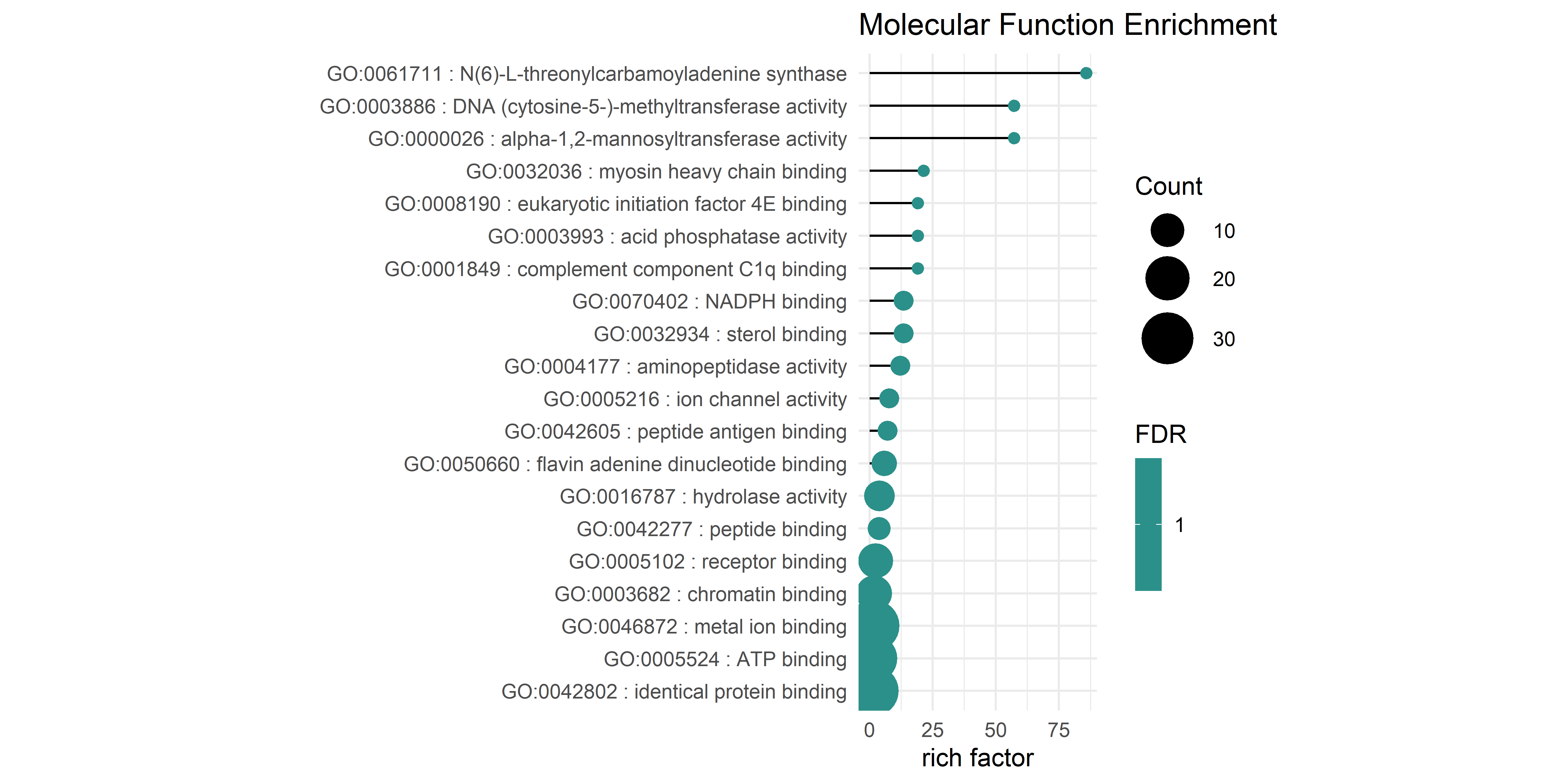

Supplement: Supplementary file 3 — Supplementary Information 3. [file 41598_2022_15861_MOESM3_ESM.zip › Singh et al_Supp Material_3_GO_analysis_Fig3/EPN.DownAndSustained.GOMF.png]

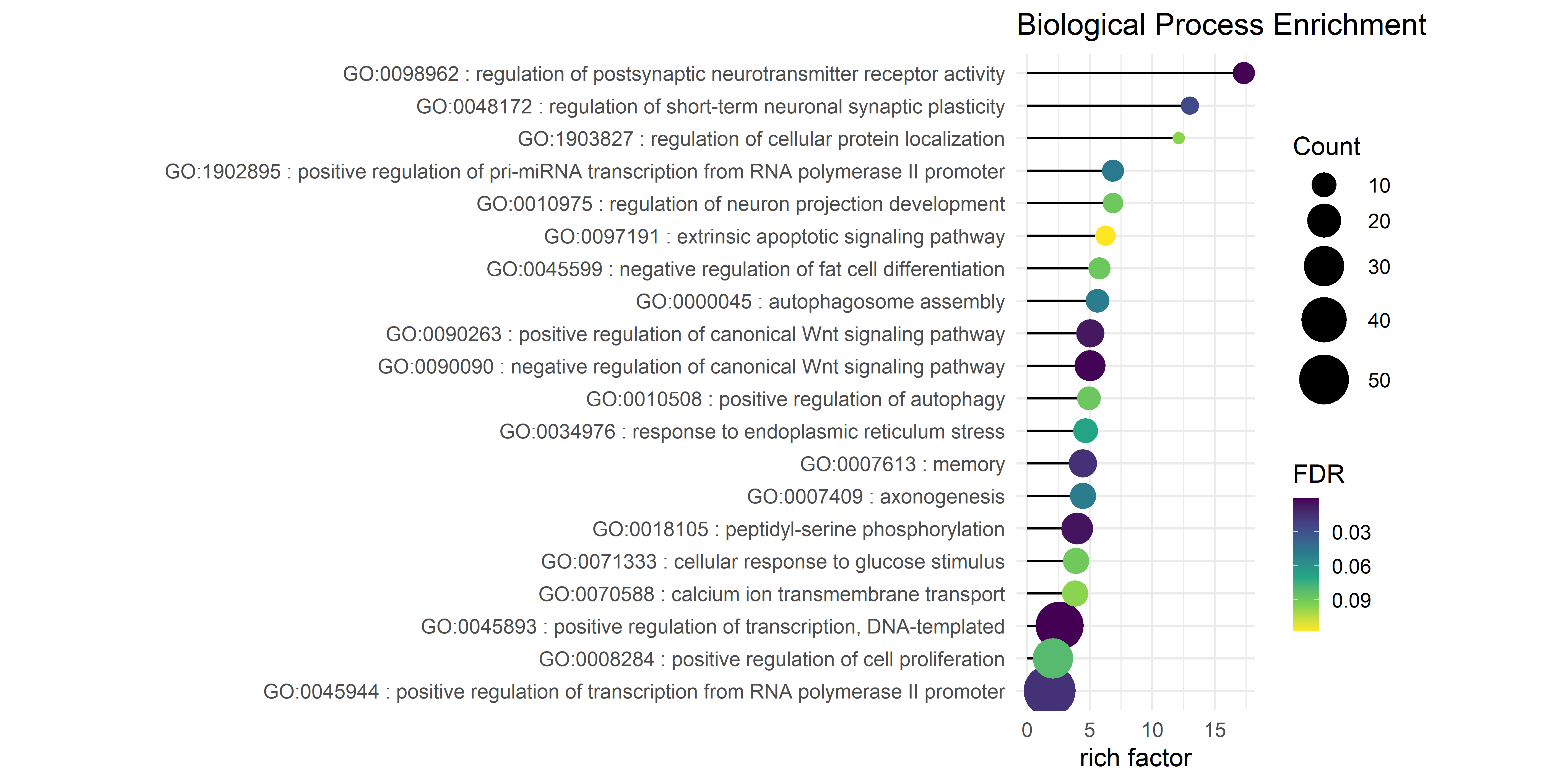

Supplement: Supplementary file 3 — Supplementary Information 3. [file 41598_2022_15861_MOESM3_ESM.zip › Singh et al_Supp Material_3_GO_analysis_Fig3/EPN.UpAndSustained.GOBP.png]

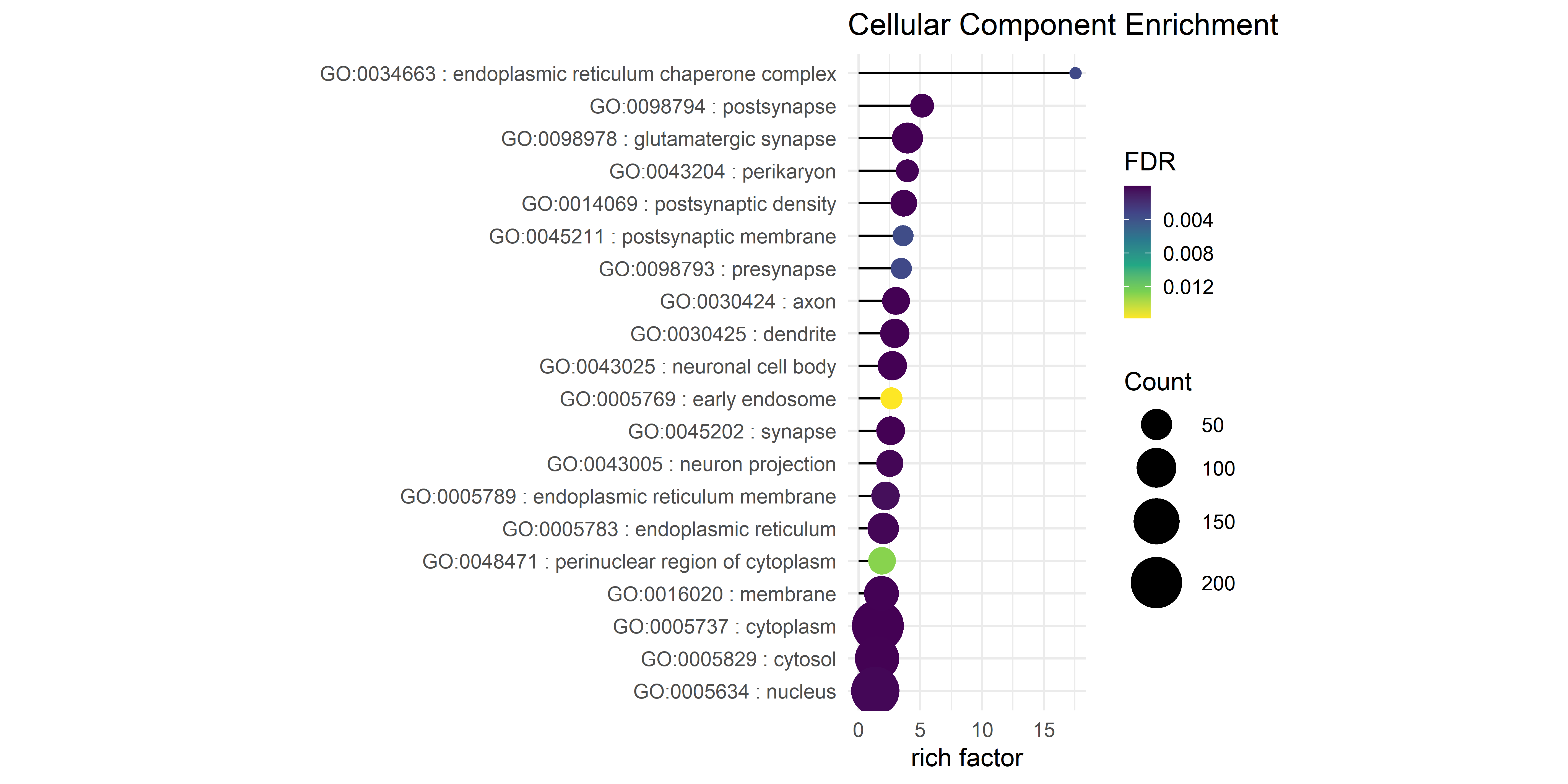

Supplement: Supplementary file 3 — Supplementary Information 3. [file 41598_2022_15861_MOESM3_ESM.zip › Singh et al_Supp Material_3_GO_analysis_Fig3/EPN.UpAndSustained.GOCC.png]

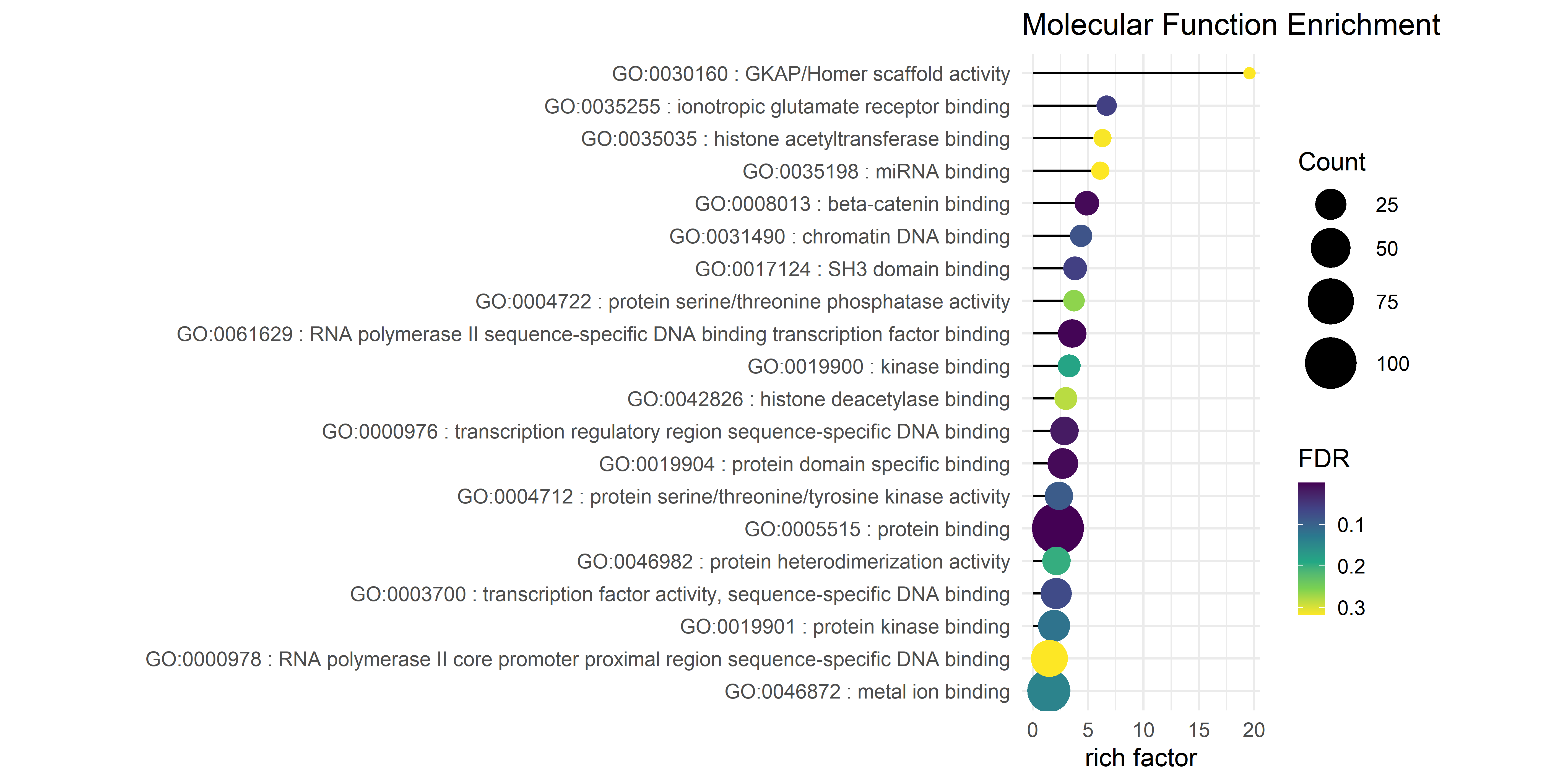

Supplement: Supplementary file 3 — Supplementary Information 3. [file 41598_2022_15861_MOESM3_ESM.zip › Singh et al_Supp Material_3_GO_analysis_Fig3/EPN.UpAndSustained.GOMF.png]
